# Antagonistic bacteria disrupt calcium homeostasis and immobilize algal cells

**DOI:** 10.1038/s41467-017-01547-8

**Published:** 2017-11-24

**Authors:** Prasad Aiyar, Daniel Schaeme, María García-Altares, David Carrasco Flores, Hannes Dathe, Christian Hertweck, Severin Sasso, Maria Mittag

**Affiliations:** 1 Institute of General Botany and Plant Physiology, Friedrich Schiller University, Am Planetarium 1, 07743 Jena, Germany; 2 Leibniz Institute for Natural Product Research and Infection Biology (HKI), Beutenbergstr. 11 a, 07745 Jena, Germany

## Abstract

Photosynthetic unicellular organisms, known as microalgae, are key contributors to carbon fixation on Earth. Their biotic interactions with other microbes shape aquatic microbial communities and influence the global photosynthetic capacity. So far, limited information is available on molecular factors that govern these interactions. We show that the bacterium *Pseudomonas protegens* strongly inhibits the growth and alters the morphology of the biflagellated green alga *Chlamydomonas reinhardtii*. This antagonistic effect is decreased in a bacterial mutant lacking orfamides, demonstrating that these secreted cyclic lipopeptides play an important role in the algal–bacterial interaction. Using an aequorin Ca^2+^-reporter assay, we show that orfamide A triggers an increase in cytosolic Ca^2+^ in *C*. *reinhardtii* and causes deflagellation of algal cells. These effects of orfamide A, which are specific to the algal class of Chlorophyceae and appear to target a Ca^2+^ channel in the plasma membrane, represent a novel biological activity for cyclic lipopeptides.

## Abstract (web summary)

Predatory or competitive interactions between microbes are poorly understood but likely influence global nutrient cycles. Here, the authors show that Pseudomonas bacteria could immobilize algal cells, potential prey, by releasing secondary metabolites that induce a Ca^2+^ signal and algal deflagellation.

## Introduction

Carbon fixation by photosynthetic organisms is a crucial step in the global carbon cycle, converting CO_2_ and light into valuable, energy-rich organic molecules. Apart from higher plants, prokaryotic and eukaryotic microalgae in aquatic environments are responsible for approximately 50% of all carbon fixation annually^1^. In addition, these photosynthetic microorganisms are at the base of aquatic food webs, thus playing a key role in diverse ecosystems. In their freshwater and marine habitats, microalgae also naturally coexist with a large variety of other microorganisms. In analogy to the terrestrial plant environment, these complex interactions may influence the fitness and performance of the microalgae and even lead to their death. However, compared to the large body of knowledge on the effects of mutualists or parasites on higher plants^2^, there is limited information on biotic interactions of photosynthetic microbes. Only recently, an increasing number of studies were reported on the characterization of algicidal bacteria and of natural products or enzymes that directly affect algal fitness^3–7^. In most cases, diffusible algicidal agents are secreted by the bacteria that inhibit cell growth, disrupt the cell envelope, and/or rapidly lyse target cells^7^. Other algicidal bacteria require direct contact with the algae to exert their destructive effects. In these cases, they employ enzymes to cleave polysaccharides or proteins that are present on the cell wall of the algae, thereby disrupting cell integrity and causing lysis^7^. However, most algicidal factors, their effects, and the involved signaling pathways remain to be elusive^8^. This lack of knowledge is surprising in light of the well-known relevance of microalgae for life on Earth and their emerging importance for biofuel production.

The poor knowledge on the mediators of microalgal–microbial communities may—at least in part—be attributable to the lack of interacting species that are genetically tractable. To evaluate the factors governing the interaction between bacteria and microalgae, we thus focused on a fully sequenced model organism, *Chlamydomonas reinhardtii*, for which molecular tools are available^9–11^. This biflagellate unicellular green alga is ubiquitously distributed in habitats such as fresh water and moist soil, and has been extensively used to study light perception, photosynthesis, and flagellar function, and also with regard to human diseases^9, 12, 13^. However, *C*. *reinhardtii* is usually grown axenically in the laboratory, and only a few studies have explored how this algal genus responds to changes in biotic factors^14–16^. Here, we report on the biological function of secondary metabolites from the bacteria *Pseudomonas protegens* in their interplay with the motile microalga *C*. *reinhardtii*. *P*. *protegens* (formerly known as *P*. *fluorescens*)^17^ also lives in aquatic and soil environments, where it either promotes plant growth^18^ or leads to the cell death of the photosynthetic organism^3^. By means of high-resolution mass spectrometry and a tailor-made reporter system, we found that *P*. *protegens* employs chemical mediators, including cyclic lipopeptides to deflagellate the *C*. *reinhardtii* cells and alter cytosolic Ca^2+^ levels. Immobilization and disruption of algal cells by *P*. *protegens* and its secondary metabolites appears to provide an advantage to the bacteria when deprived of micronutrients.

## Results

### *P*. *protegens* Pf-5 arrests the growth of *C*. *reinhardtii*

**Fig. 1:**
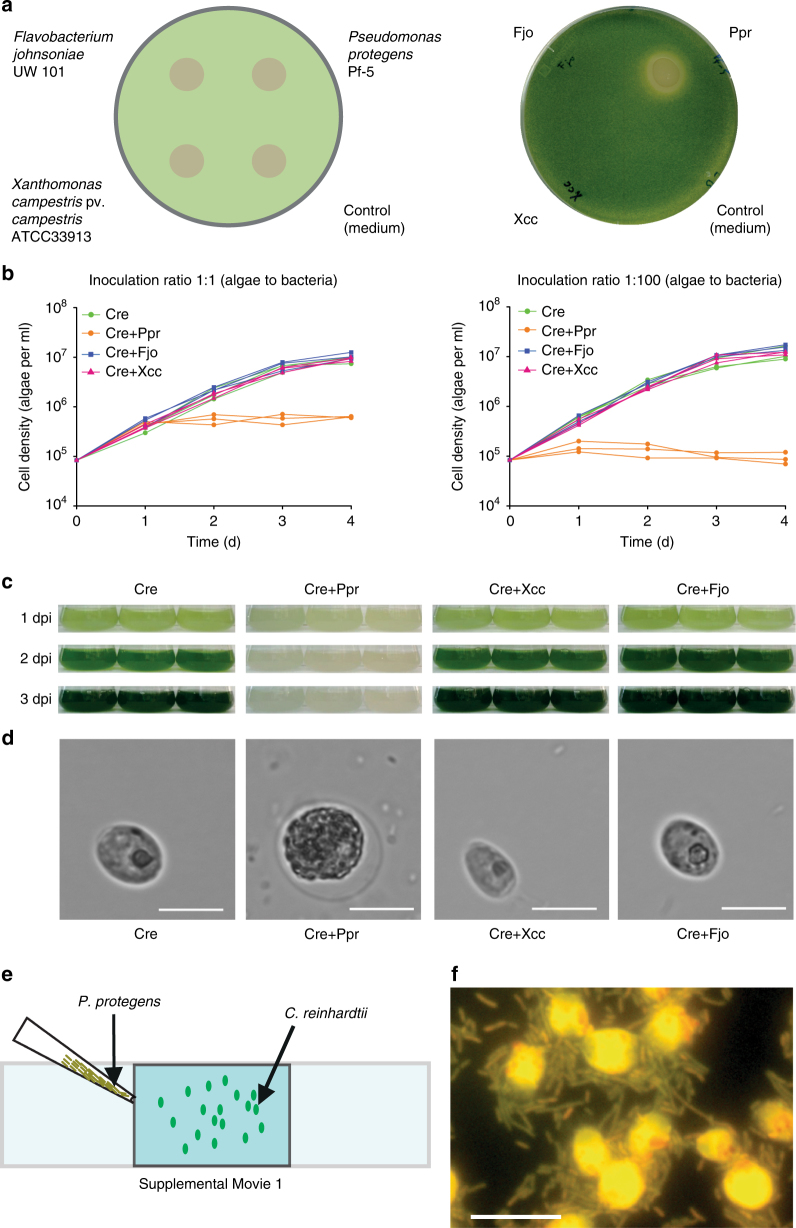
*P*. *protegens* swarms to *C*. *reinhardtii* and leads to growth arrest and changes in algal morphology. **a** Co-cultivation of *C*. *reinhardtii* and bacteria on agar plates after 3 days reveals the inhibitory effect of *P*. *protegens*. Suspensions of different bacteria were applied to restricted areas of a plate that contains *C*. *reinhardtii* within a top layer of TAP agar. LB medium was used as control. **b** Liquid co-cultivation at indicated ratios of algae to bacteria used for inoculation shows algal growth arrest in the presence of *P*. *protegens*. Cultures were inoculated to obtain initial cell densities of 8.3 × 10^4^ algae per ml, and in coculture 8.3 × 10^4^ (1:1) or 8.3 × 10^6^ (1:100) bacteria per ml were added. Values of triplicate cultures are shown. **c** Photographs of the experiment depicted in **b** (1:100 ratio) show algal growth arrest by the color change. **d** Morphology change of algal cells in the presence of *P*. *protegens* after 24 h in mixed culture as compared to an axenic culture by bright-field microscopy using a magnification of ×630. Scale bar: 10 µm. **e** *P*. *protegens* swarms around the algal cells and surrounds them within minutes. Scheme depicting the experimental procedure used to visualize live interaction of *P*. *protegens* with *C*. *reinhardtii* (Supplementary Movie 1). Overall, 10 µl of an overnight culture of *P*. *protegens* in LB medium were introduced from one corner of the coverslip as shown in the scheme. **f** Cells of *C*. *reinhardtii* and *P*. *protegens* were immobilized after 10 min in coculture on a coated glass slide and stained with acridine orange. Cells were visualized using fluorescence microscopy at ×1000 magnification. Scale bar: 10 µm. Cre *Chlamydomonas reinhardtii*, Fjo *Flavobacterium johnsoniae*, Xcc *Xanthomonas campestris* pv. *campestris*, Ppr *Pseudomonas protegens*, dpi days post inoculation. **a**–**e** All experiments were performed in three biological replicates and representative pictures (**d**) and a representative Supplementary Movie 1 are shown. **f** shows a representative section of an acridine orange-stained sample as treated in **e**, but for 10 min. The experiments were replicated three (**a**) or two times (**e**) or performed once (**b**, **d**)

To investigate whether heterotrophic bacteria sharing the same habitat as *C*. *reinhardtii* affect algal growth, we selected *Flavobacterium johnsoniae*, *Xanthomonas campestris* pv. *campestris*, and *P*. *protegens*. Specifically, we used sequenced strains of bacterial species previously isolated from a microalgal culture^14^ and applied the bacteria to a restricted area on a plate that contains *C*. *reinhardtii* cells (Fig. 1a). In coculture with *F*. *johnsoniae* or *X*. *campestris*, the growth of *C*. *reinhardtii* appeared to be unaffected, i.e., comparable to that on the medium control. In contrast, *P*. *protegens* strongly inhibited the growth of *C*. *reinhardtii*. Likewise, in liquid cultures (inoculation ratio of 1:1 and 1:100 algae to bacteria), only *P*. *protegens* substantially decreased the cell density of *C*. *reinhardtii* compared to pure algal cultures (Fig. 1b). Algal growth was stopped within the first day in coculture (1:100 ratio) or starting 1 day after inoculation (1:1 ratio). Photographs taken of the cultures in a replete medium show that the typical green color of the algal culture is absent when cocultivated with *P*. *protegens* (1:100 ratio) (Fig. 1c), indicating the arrest of the algal growth. Furthermore, in contrast to the other bacteria, *P*. *protegens* altered the morphology of the algal cells within 1 day in coculture. The usually oval algal cells were enlarged and almost circular, and their inner structure became granular (Fig. 1d).

It is known that the depletion of micronutrients such as iron or zinc can affect the biocontrol properties of *P*. *protegens* ^19, 20^. To test whether *P*. *protegens* may benefit from *C*. *reinhardtii* under nutrient-limiting conditions, we omitted the trace elements (Fe, Zn, Cu, Co, Mn, and Mo) from the growth medium. While bacterial growth was largely unaffected in a replete medium (Supplementary Fig. 1a, b), it was enhanced in coculture in a medium lacking micronutrients, as compared to the axenic bacterial culture grown in the same medium (Supplementary Fig. 2a). Algal cells also showed an altered morphology after 1 day in coculture in the medium depleted of micronutrients, and they had lost their flagella (Supplementary Fig. 2b).

To learn more about the dynamics of the interaction of *C*. *reinhardtii* with *P*. *protegens*, we recorded a video (Supplementary Movie 1). Initially, the algal cells swim freely in the medium. After 19 s, the bacteria were introduced from one corner of the slide (Fig. 1e). Within only 1.5 min, the bacteria start to surround the algal cells that already appear to be immobilized. Two minutes later, the intensity of the swarming reaches a maximum, and algal cells are completely encircled by bacteria. The physical contact of the bacteria and the algae was independently confirmed using an acridine orange-stained fluorescent micrograph after a 10-min incubation time (Fig. 1f).

### Orfamide A immobilizes several Chlorophyceae

**Fig. 2:**
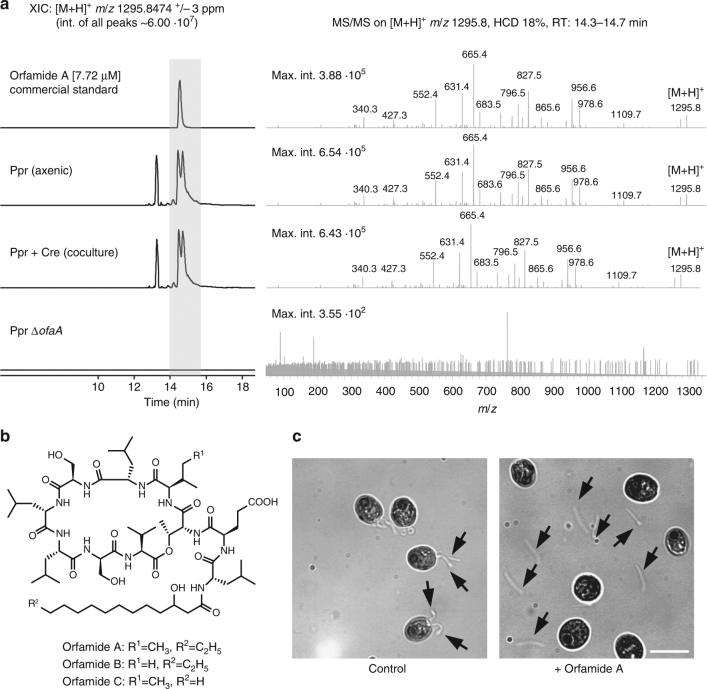
Orfamide A is released by *P*. *protegens* and leads to deflagellation of the algal cells. **a** Detection of orfamide A by high-resolution liquid chromatography-tandem mass spectrometry (LC–HRMS/MS). Extracted-ion chromatograms (XIC) of orfamide A (C_64_H_114_N_10_O_17_, accurate mass 1294.836345 u) [M+H]^+^ *m*/*z* 1295.8474 ±3 ppm and its MS/MS fragmentation pattern are shown for orfamide A commercial standard and different extracts from culture supernatants including an extract of *P*. *protegens* in axenic culture, an extract of *P*. *protegens* in coculture with *C*. *reinhardtii*, and an extract of *P*. *protegens ΔofaA* in axenic culture (orfamide deletion mutant). In MS/MS spectra, only the first decimal of *m*/*z* values is shown to improve figure legibility. One independent experiment, with one biological replicate and two technical replicates was conducted. **b** Structures of orfamide A, B, and C. **c** Orfamide A deflagellates *C*. *reinhardtii*. Wild-type cells of *C*. *reinhardtii* were grown in TAP medium. A drop of 20 µl culture was applied on the slide. The cells were visualized in a bright-field microscope at ×200 magnification (scale bar: 10 µm); the cell movement was recorded before and after application of 35 µM orfamide A or methanol as control (Supplementary Movies 2–5). To visualize cells that retained or lost their flagella, cells were treated with orfamide A or methanol (control) for 30 s, fixed with 8% potassium iodide and visualized using differential interference contrast (DIC) microscopy at ×630 magnification. The orfamide and control treatments were done in biological triplicates (see also Supplementary Movies 2–5); a representative picture is shown. The experiment was replicated twice

*P*. *protegens* biosynthesizes a wide range of natural products, including medium-size molecular weight compounds such as polyketides and nonribosomal peptides^21^ that could have a role in the bacterial–algal interplay. To study the chemical mediators of the interaction, we monitored two-dimensional patterns of diffusible metabolites between mixed bacterial and algal cultures by MALDI-imaging mass spectrometry (MALDI-IMS)^22–24^. Organisms were directly grown on disposable indium tin oxide-coated glass slides that were overlaid with a layer of solidified growth medium (Supplementary Fig. 3a). These experiments revealed a cluster of very intense ions, in the range of *m*/*z* 1300–1360, localized mainly to the area surrounding the *P*. *protegens* growth (Supplementary Figs. 3b and 4). To identify these ions, we analyzed extracts from culture supernatants of *P*. *protegens* grown axenically and in coculture with *C*. *reinhardtii* by liquid chromatography–high-resolution tandem mass spectrometry (LC–HRMS/MS). The ions detected by MALDI-IMS were also found in the extracts by LC–HRMS, and their exact masses fitted to the natural products orfamides (Fig. 2a; Supplementary Fig. 5). We used the commercial standard of orfamide A as a reference and a *P*. *protegens* Δ*ofaA* mutant lacking all orfamides^25^ to confirm the identity of orfamide A (Fig. 2a). Orfamides B and C were also detected in the extracts (Supplementary Fig. 5). Moreover, the extracted-ion chromatogram of *m*/*z* 1295.8474 ±3 ppm (orfamide A [M+H]^+^) showed two additional chromatographic peaks at different retention times compared to orfamide A (even when the mass tolerance window was reduced to 1 ppm), suggesting the presence of undescribed isomers of orfamide A in the extracts (Fig. 2a).

We next analyzed whether orfamide A (Fig. 2b) alone has an effect on the algal cells. A drop of *C*. *reinhardtii* cell suspension was placed on a glass slide, and a video was recorded before adding orfamide A or methanol (negative control), which were carefully applied from the top (Supplementary Movies 2–5). Whereas no effect was observed for the negative control, upon the addition of orfamide A, the cells stopped moving within 30–40 s. Light micrographs from orfamide A treated and untreated cells revealed that exposure to orfamide A results in the rapid deflagellation of algal cells (Fig. 2c). These results suggest that the bacteria employ orfamide A as a tool to incapacitate the algal cells, and thus the algal cells lose the ability to evade the bacteria by swimming away.

The specificity of orfamide A-triggered loss of motility was further tested with flagellated algae other than *C*. *reinhardtii*. Therefore, a drop of algal cell suspension was placed in each case on a glass slide, and the cells were visualized using bright-field microscopy. A video was recorded before and after adding orfamide A. Negative controls with methanol that were carried out as well did not influence motility in any case (not shown). Other Chlorophyceae, such as the closely related marine *Chlamydomonas* sp. SAG 25.89 (Supplementary Fig. 6), *Haematococcus pluvialis* of the order of Chlamydomonadales, as well as the colony-forming *Gonium pectorale* of the order of Volvocales lost motility to a large extent upon orfamide A treatment (Table 1; Supplementary Movies 6–11). In contrast, *Pedinomonas minor*, a Chlorophyte more distantly related to *C*. *reinhardtii* from the class of Pedinophyceae as well as the Euglenophyte, *Euglena gracilis*, retained motility upon orfamide A exposure (Table 1; Supplementary Movies 12–15). These data indicate that orfamide A specifically affects the motility of algae from the Chlorophyceae, whereas tested algae outside this taxonomic class are not vulnerable to this bacterial secondary metabolite.

**Table 1:** Loss of motility of exemplary flagellate algae upon treatment with orfamide A

Species	Class	Phylum	Habitat	Loss of motility	Supplementary videos
*Chlamydomonas reinhardtii*	Chlorophyceae	Chlorophyta	FW/WS	Yes	2–5
*Chlamydomonas* sp.	Chlorophyceae	Chlorophyta	Marine	Yes	6, 7
*Haematococcus pluvialis*	Chlorophyceae	Chlorophyta	FW/WS	Yes	8, 9
*Gonium pectorale*	Chlorophyceae	Chlorophyta	FW/WS	Yes	10, 11
*Pedinomonas minor* ^a^	Pedinophyceae	Chlorophyta	FW/WS	No	12, 13
*Euglena gracilis*	Euglenophyceae	Euglenophyta	FW/WS	No	14, 15

*FW* Freshwater, *WS* wet soil

Orfamide A was applied at a concentration of 35 µM

^a^Non-axenic strain

### Establishment of an aequorin reporter in *C*. *reinhardtii*

**Fig. 3:**
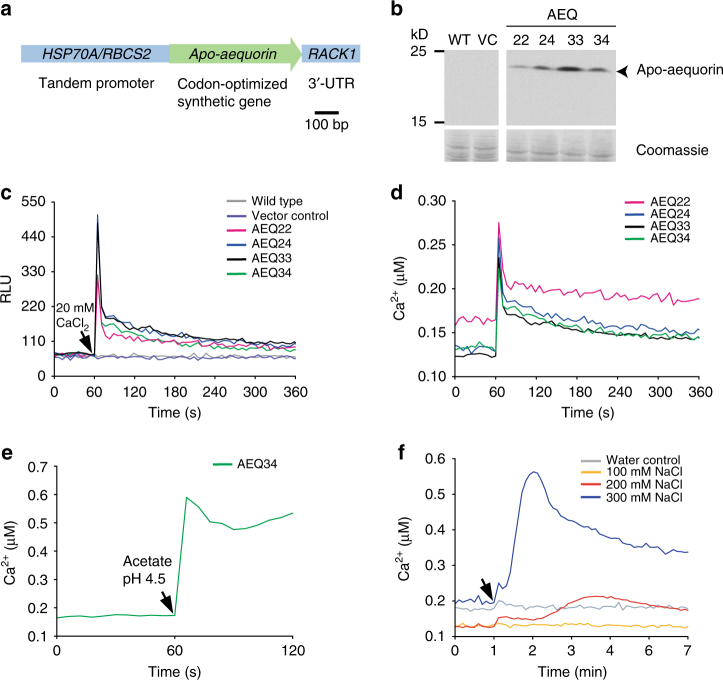
Establishment of an aequorin-based calcium assay in *C*. *reinhardtii* and its applicability for abiotic stresses. **a** The apo-aequorin expression cassette includes a codon optimized gene and *cis*-acting elements for efficient expression (Supplementary Fig. 7). **b** Immunoblot showing the expression of apo-aequorin (22 kD) in transgenic lines AEQ22, AEQ24, AEQ33, and AEQ34 compared to wild type (WT) and a vector control (VC). As a loading control, selected protein bands from the Coomassie-stained PVDF membrane are shown. Another biological replicate showed qualitatively identical results. **c**, **d** External calcium triggers luminescence in aequorin-expressing lines. After measuring the resting Ca^2+^ concentration in relative light units (RLU), 20 mM of external CaCl_2_ were added as indicated by the black arrowhead. Obtained RLU values (**c**) were converted to relative Ca^2+^ concentrations (**d**) by triggering maximal luminescence (Supplementary Fig. 8). **e** Acidification of the medium causes a prompt increase in cytosolic Ca^2+^ ions. After measurement of the resting Ca^2+^ concentration, sodium acetate buffer pH 4.5 was added to a final concentration of 20 mM (black arrowhead) to AEQ34 cells in HEPES buffer pH 7.4. **f** Salt stress results in an increase in Ca^2+^ ions. Coelenterazine loaded cells of transgenic aequorin reporter line AEQ34 were treated with increasing concentrations of NaCl (black arrowhead) as indicated. As a control, an equivalent volume of deionized water was used. **c**–**f** Each line in the graphs represents the mean of three biological replicates and each biological replicate includes three technical replicates. All experiments were replicated twice except for **e**, which was done once

Since it is known that Ca^2+^ spikes can cause flagellar excision in *C*. *reinhardtii* ^26^, we aimed at monitoring cytosolic Ca^2+^ changes in situ during the microbial interaction. For this purpose, we established an aequorin reporter system in *C*. *reinhardtii*. We constructed a gene cassette containing the *apo-aequorin* gene of *Aequoria victoria* ^27^, which was codon optimized for efficient expression in *C*. *reinhardtii*, and introduced it into the algal cells (Fig. 3a and Supplementary Fig. 7). Several transgenic lines (AEQ22, AEQ24, AEQ33, and AEQ34) expressed the 22-kD apo-aequorin successfully (Fig. 3b) and were selected for further examination.

As high extracellular Ca^2+^ concentrations reportedly cause an increase of cytosolic Ca^2+^ levels^28^, we initially analyzed aequorin activities by providing external Ca^2+^ to the AEQ cell lines. Notably, upon addition of excess of external Ca^2+^, all four transgenic AEQ lines immediately showed a luminescence spike (Fig. 3c). This response was neither observed in the wild type nor in the negative control containing the vector only. In order to estimate the intracellular Ca^2+^ concentrations, cell lysis was performed at the end of the measurement using an ethanol-containing saturating solution of Ca^2+^ to trigger the maximum luminescence (Supplementary Fig. 8; Fig. 3d). We also compared maximum luminescence values dependendent on the age of the cells, which was the highest during the exponential growth phase (Supplementary Fig. 9). To further validate our reporter system, abiotic stimuli (abrupt acidification^29^ and salt stress^30^) previously demonstrated to increase intracellular Ca^2+^ levels were applied to the transgenic strains. In both cases, the luminescence increased as expected (Fig. 3e, f).

### Orfamides trigger a Ca^2+^ signal and influence algal growth

**Fig. 4:**
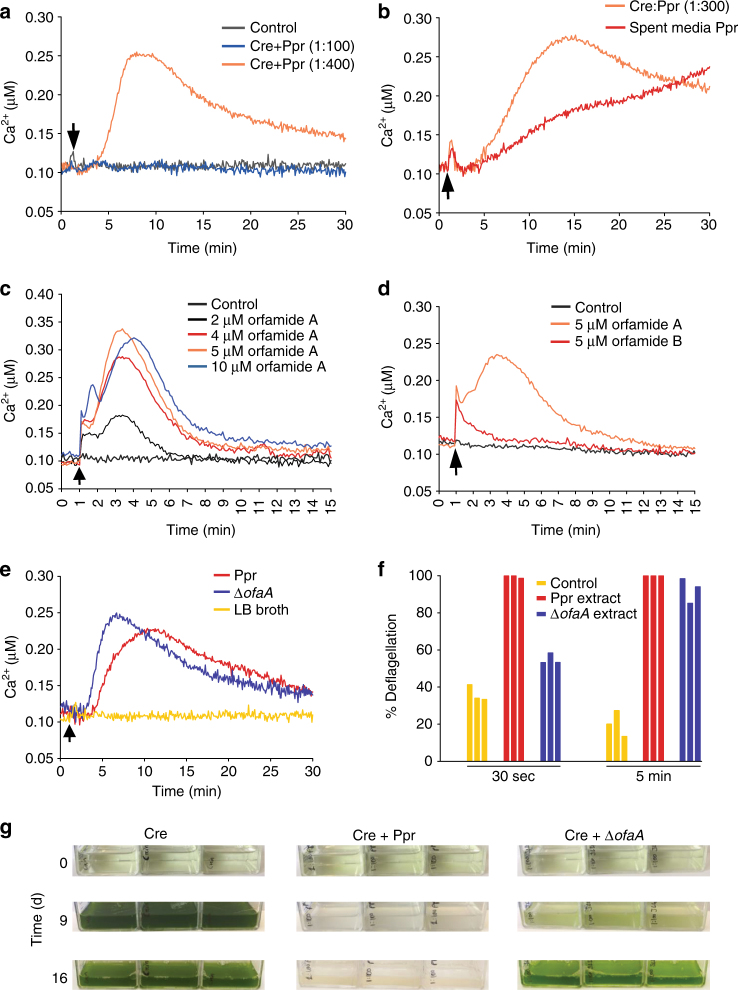
Orfamides cause a rapid increase in cytosolic Ca^2+^ and contribute to deflagellation and algal growth arrest. **a** *P*. *protegens* is able to trigger Ca^2+^ changes in *C*. *reinhardtii*. Time course of cytosolic Ca^2+^ concentrations after addition of bacteria to AEQ34 cells. For the Ca^2+^ measurements, the bacterial cell density was adjusted to the indicated cell ratios. As control, LB broth was added (black arrowhead). **b** Effect of *P*. *protegens* spent medium on cytosolic Ca^2+^ levels in *C*. *reinhardtii*. Aequorin-expressing cells (AEQ34) were incubated with *P*. *protegens* in a ratio of 1:300 and used for the measurement. Further, sterile-filtered spent medium from an overnight culture of *P*. *protegens* (black arrowhead) was used. **c** Orfamide A elicits dose-dependent Ca^2+^ changes. Orfamide A that was dissolved in methanol and further diluted in TAP medium was added to cells at the indicated concentrations. As control, methanol proportional to that of the highest concentration of orfamide A was used. **d** Ca^2+^ measurement upon treatment with 5 μM orfamide A or B. As control, methanol was used. **e** Ca^2+^ measurements with wild-type *P*. *protegens* or Δ*ofaA* mutant at a ratio of 1:400 of algae to bacteria. **f** Extracts prepared from supernatants of *P*. *protegens* and the Δ*ofaA* mutant cultures, respectively, having similar cell densities (around 3.14 × 10^8^ and 3.16 × 10^8 ^cells ml^−1^, respectively) were incubated with *C*. *reinhardtii* at a 1% concentration for 30 s or 5 min in triplicates. A 1% extract of TAP medium was taken as control. **g** Liquid co-cultivation of *C*. *reinhardtii* together with wild-type *P*. *protegens* or Δ*ofaA* mutant compared to axenic algal cultures. A 1:100 ratio of algae to bacteria was used for inoculation with an initial concentration of 10^5^ algal cells per ml. The corresponding growth curves are depicted in Supplementary Fig. 12. **a**–**e** Each line in the graph represents the mean of three biological replicates, and each biological replicate includes three technical replicates. All experiments were replicated twice except for **f** that was performed once with one biological and three technical replicates. The experiment in **g** was performed twice with three biological replicates

With a reliable aequorin reporter system at hand, we next studied the biotic interactions of *C*. *reinhardtii*. The Ca^2+^ response in aequorin-expressing *C*. *reinhardtii* cells was measured in the presence of *P*. *protegens* cells at different ratios (algae:bacteria 1:100 and 1:400). At a 1:100 ratio, no changes in cytosolic Ca^2+^ levels could be observed within the first 30 min of incubation (Fig. 4a), whereas a strong Ca^2+^ signal occurred at a ratio of 1:400 after just 5 min. These results show that the interaction between *C*. *reinhardtii* and *P*. *protegens* cells results in a very fast alteration of the intracellular Ca^2+^ level, once a critical number of bacterial cells surrounding the algae (Supplementary Movie 1) is reached. In contrast, when adding *X*. *campestris* or *F*. *johnsoniae* cells at the same ratios, no Ca^2+^ release was triggered (Supplementary Fig. 10a and b). Even a 4500-fold excess of *X*. *campestris* or *F*. *johnsoniae* did not trigger a Ca^2+^ signal, demonstrating that the response of *C*. *reinhardtii* is not generally caused by the presence of bacteria, but it is specific to *P*. *protegens*.

We also found that a direct contact of *P*. *protegens* with the algal cells is not required to trigger the Ca^2+^ release. The cell-free supernatant of the bacterial culture also caused a signal in the aequorin reporter line (Fig. 4b), thus showing that a diffusible signal is responsible for the Ca^2+^ release. Next, we tested the capability of orfamide A to cause alterations in cytosolic Ca^2+^ in a manner similar to the coculture and the supernatant of *P*. *protegens*. Solutions of pure orfamide A at different concentrations were added to the algal aequorin reporter line. Whereas the control with the solvent alone showed no alteration, we observed a dose-dependent change in the Ca^2+^ levels up to ~5 µM orfamide A within the first 5 min after the addition of orfamide A. Higher levels of orfamide A (10 µM) resulted in saturation (Fig. 4c). These data unequivocally show that orfamide A affects Ca^2+^ homeostasis in the bacterial–microalgal interaction. In addition, the concentration-dependent response up to ca. 5 µM orfamide A mirrors the effect of the bacterial cell density when surrounding the algae, thus causing a high orfamide A concentration in the vicinity of the microalgae.

The extended response of *C*. *reinhardtii* to the spent media of *P*. *protegens* (Fig. 4b) suggested that chemical mediators other than orfamide A may also contribute to the signal. As orfamide B was also identified in *P*. *protegens* (Supplementary Fig. 5) and is commercially available, we analyzed if it has a similar effect as orfamide A. We observed that orfamide B triggers a Ca^2+^ release, whose signature, however, differs from that of orfamide A (Fig. 4d). A sharp peak of low amplitude observed immediately after the addition of orfamide A was also present with orfamide B, but the larger and broader response found with orfamide A was missing. Yet, orfamide B deflagellates the algal cells (Supplementary Fig. 11).

Orfamides are biosynthesized from an operon consisting of three structural genes known as *ofaA*, *ofaB*, and *ofaC*, encoding nonribosomal peptide synthetases^31, 32^. To investigate the *in vivo* role of orfamides in algal–bacterial mixed cultures, we used a Δ*ofaA* mutant^25^ that lacks all orfamides. The observation that this mutant is still able to elicit a Ca^2+^ signal (Fig. 4e) indicates that *P*. *protegens* produces infochemicals other than the orfamides with the capacity to influence the cytosolic Ca^2+^ levels in *C*. *reinhardtii*. However, a 1% extract from the culture supernatant of the Δ*ofaA* mutant deflagellated algal cells after 30 s only at a relatively low rate compared to the supernatant from a *P*. *protegens* culture (Fig. 4f). After a longer incubation time (5 min), the rate of deflagellation was high in both cases (Fig. 4f). These data show that the orfamides contribute to the rapid deflagellation event of algal cells. We also looked at the growth of algal cells in coculture with the mutant. Although the Δ*ofaA* mutant initially suppressed algal growth in a coculture assay, the algae recovered and started to grow after 9–10 days (Fig. 4g; Supplementary Fig. 12). In contrast, wild-type *P*. *protegens* suppressed algal growth over a period of 19 days. Although these data provide strong evidence that further secondary metabolites contribute to the complex interplay between *P*. *protegens* and the algae, they clearly suggest at the same time that the orfamides are involved in the antagonistic effect exerted by *P*. *protegens* on the algae.

### Orfamide induces channel-based influx of extracellular Ca^2+^

**Fig. 5:**
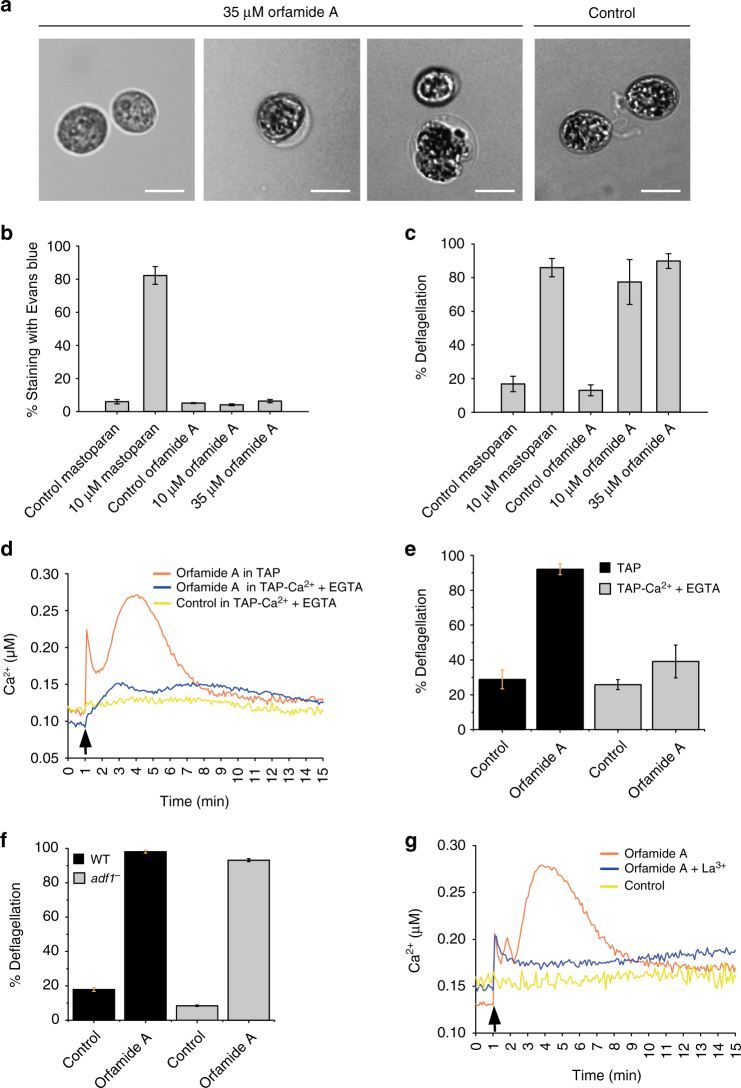
Orfamide A induces influx of extracellular Ca^2+^, probably through an unidentified channel. **a** Cell morphology of *C*. *reinhardtii* exposed to 35 µM of orfamide A after 24 h. The density of algal cells was 4 × 10^6^ cells ml^−1^. Algal cells were observed by bright-field microscopy using a magnification of ×400 (scale bar: 10 µm). **b** Evans blue staining of wild-type *C*. *reinhardtii* (strain SAG 73.72) upon treatment with orfamide A and mastoparan at the indicated concentrations. As controls, methanol or water were used at equivalent concentrations. **c** Deflagellation of wild-type *C*. *reinhardtii* upon treatment with orfamide A and mastoparan at the indicated concentrations. As control, methanol or water were used at equivalent concentrations. **d** Orfamide A triggers Ca^2+^ changes mostly from external pools. AEQ34 cells were grown in TAP medium for 72 h. Before incubation with coelenterazine, the TAP medium was replaced with TAP lacking Ca^2+^ and the cells were incubated with 50 µM EGTA for 5 minutes before commencing the Ca^2+^ measurement. As negative control, AEQ34 cells in TAP lacking Ca^2+^ were treated with methanol. **e** Deflagellation of wild-type *C*. *reinhardtii* upon treatment with 35 µM of orfamide A in TAP medium or TAP lacking Ca^2+^ (see **d**). In the latter case, the cells were washed three times with TAP lacking Ca^2+^ supplemented with 50 µM EGTA before the treatment with orfamide A. **f** Deflagellation in the *adf-1* mutant and its underlying wild type (WT, strain CC-620) upon treatment of cells with 35 µM of orfamide A. **g** Lanthanum (La^3+^) inhibits orfamide-induced Ca^2+^ signaling. Overall, 20 µM LaCl_3_ were added to the cells in HEPES buffer, pH 7.4, 1 min before the Ca^2+^ measurement was started. In **b**, **c**, **e**, and **f**, all experiments were performed with three biological replicates and were replicated twice (**b**, **c**, **f**) or once (**e**). Error bars represent the standard deviation. In **d** and **g**, each line in the graph represents the mean of three biological replicates, and each biological replicate includes three technical replicates. Except for **a**, which was performed once, all experiments were replicated twice. In **a**, representative pictures are shown

In the next step, we focused further on the effect of orfamide A and examined if it is able to alter the morphology of *C*. *reinhardtii*, as observed after 24-h incubation of the algae with *P*. *protegens* (Fig. 1d). Exposure of algal cells to 35 µM orfamide A resulted in deflagellation, as described before, but the oval shape of the cells was largely unaffected (Fig. 5a, left image). In a few cases, a circular and enlarged morphology was also visible after 24 h (Fig. 5a, medium and right images) that was not observed in the control. These data show that orfamide A has the capability to alter the morphology and suggest that a coordinated action of orfamides as well as of other chemical compounds is needed for large-scale changes in morphology after 24 h.

We further checked if the deflagellation and the increase in Ca^2+^ in the algal cells are caused by permeabilization effects by orfamide A using the vital stain Evans blue. In the positive control, 10 µM mastoparan, known as a permeabilization agent^33^, leads to an efficient staining of approximately 80% of the *Chlamydomonas* cells with Evans blue (Fig. 5b) and concomitant high rates of deflagellation (Fig. 5c). In contrast, 35 µM (used in Fig. 2c) as well as 10 µM orfamide A, which still causes ~80% of deflagellation (Fig. 5c) did not result in an increased staining of *Chlamydomonas* cells compared to the control (Fig. 5b). Algal cells treated with 10 µM orfamide B did not show increased permeability to Evans blue either (Supplementary Fig. 11). These data show that orfamides do not provoke a major permeabilization of the algal cells—at least not on a short term—for orfamide-induced deflagellation in Chlorophyceae as observed with orfamide A (Table 1, see Discussion).

To investigate whether the orfamide-mediated rise in cytosolic Ca^2+^ is due to an influx of Ca^2+^ from the extracellular medium or if it is released from intracellular Ca^2+^ stores, we resuspended the cells in a medium lacking Ca^2+^ ions that had been additionally supplemented with 50 µM EGTA to sequester any possibly existing residual Ca^2+^ ions. Upon exposure of the cells to orfamide A, we observed only a very small increase in Ca^2+^ in this case, indicating that the orfamide-triggered rise in Ca^2+^ requires an influx of Ca^2+^ from outside the cell (Fig. 5d). We also investigated the link between the presence of Ca^2+^ in the medium, the action of orfamide A, and deflagellation. For this purpose, we examined the rate of orfamide-induced deflagellation in *C*. *reinhardtii* in a medium with or without Ca^2+^. Clearly, orfamide A-based deflagellation depends on the presence of Ca^2+^ in the medium (Fig. 5e). These findings link the Ca^2+^ uptake caused by the presence of orfamide A and consequently triggered deflagellation, pointing to the cytosolic Ca^2+^ elevation as the trigger for deflagellation.

In the next step, we used a mutant known as *adf-1*, which encodes for a Ca^2+^ channel shown to be responsible for deflagellation via influx of Ca^2+^ upon acid shock. The involved Ca^2+^ channel was recently identified as TRP15^29, 34^. The *adf-1* mutant was efficiently deflagellated upon orfamide A treatment (Fig. 5f), suggesting that the TRP15 channel is not involved (or is at least not the only Ca^2+^ channel) in the orfamide A signaling pathway. If the Ca^2+^ channel blocked in the *adf-1* mutant was the sole channel mediating the Ca^2+^ influx, orfamide A would not cause a significant increase in Ca^2+^ in this mutant and the mutant would not deflagellate. In contrast, if another Ca^2+^ channel of *Chlamydomonas* is involved, deflagellation would occur.

To test this possibility, we used lanthanum (La^3+^), a well-known inhibitor of Ca^2+^ channels^29, 35^. The addition of orfamide A in the presence of La^3+^ resulted in a massively reduced level of Ca^2+^ compared to the absence of La^3+^ (Fig. 5g). Altogether, our results indicate that orfamide A from *P*. *protegens* induces the influx of extracellular Ca^2+^ through an unidentified Ca^2+^ channel (other than TRP15) in the algal plasma membrane.

## Discussion

Ca^2+^ signaling has been implicated in early stages of higher plant–microbe interactions of both symbiotic and antagonistic nature^36^. Likewise, in marine diatoms, environmental signals and intraspecies interactions can activate Ca^2+^ signaling^37, 38^. By means of an aequorin reporter line, we have demonstrated that bacteria substantially perturb the cytosolic Ca^2+^ levels in *C*. *reinhardtii* at different time scales, within minutes (Fig. 4a) to hours (Supplementary Fig. 13). The changes in Ca^2+^ do not necessarily result in deflagellation of a *C*. *reinhardtii* cell, but are also involved in light-signaling pathways from the eyespot to the flagella, thus controlling algal movements^39^. Similarly, elevated salt concentrations evoke an increase in Ca^2+^, but not deflagellation^30^. The strong rise in Ca^2+^ in response to acidification and salt stress^26, 29, 30^ was confirmed with the aequorin assay in this study (Fig. 3e, f), and a rapid increase in Ca^2+^ is also evident upon orfamide A and B treatment (Fig. 4d).

Experiments with calcium-sensitive dyes have shown that localization of Ca^2+^ primarily in the apical part of the algal cell and the timing of the Ca^2+^ signals are important factors in acid-induced deflagellation^26^. In agreement with this regulatory role of Ca^2+^ signals in deflagellation, we observed that both orfamide-induced Ca^2+^ elevation (Fig. 5d) and deflagellation (Fig. 5e) depend on the presence of extracellular Ca^2+^ ions. Although stresses often elicit Ca^2+^ responses in *C*. *reinhardtii* that can lead to deflagellation^29, 30^, our results point to a more specific role of orfamides. We show that orfamide A exposure results in cytosolic Ca^2+^ increase, which requires a Ca^2+^ influx from outside the cell without causing a major permeabilization of the plasma membrane (Figs. 4c and 5b, d). The fact that this Ca^2+^ signal is inhibited by La^3+^ (Fig. 5g), a Ca^2+^ channel blocker^29, 35^, suggests that orfamide A targets a Ca^2+^ channel in the algal plasma membrane. In the light of these findings, an alternative mode of action of orfamides as Ca^2+^ carriers or small Ca^2+^-selective pores that are not permeable to Evans blue seems to be less likely, but remains to be tested.

In this context, it is also of interest that *Chlamydomonas* has an unusually high number (>30 predicted by its genome) of Ca^2+^ channels, especially of the TRP type^40^, most of which have not been characterized so far. It seems likely that one of them is part of the orfamide-signaling pathway. Since the well-characterized *Chlamydomonas* TRP15 Ca^2+^ channel^34^ is not involved in orfamide A-triggered Ca^2+^ uptake (or is not the only Ca^2+^ channel involved in this process) (Fig. 5f), the target of orfamide remains to be identified. Orfamide A may bind directly to its Ca^2+^ channel target, or induce channel opening indirectly, for example, by binding to another protein or lipid component of the plasma membrane. The distinct response of orfamide B (Fig. 4d) that differs from orfamide A by only one methyl group (Fig. 2b) might indicate that orfamide B may even trigger a different pathway than orfamide A. The specificity of orfamide A in immobilizing Chlorophyceae algae, but not two species from the Pedinophyceae and Euglenophytes, respectively (Table 1), further supports a specific mechanism and a signaling role of these cyclic lipopeptides that involves specialized membrane components unique to Chlorophyceae.

The majority of characterized lipopeptides interacts with lipid components of membranes, such a daptomycin, which was recently found to interfere with fluid membrane microdomains^41^. In some cases, however, protein targets were also identified^42^. In addition to the deflagellating and Ca^2+^-eliciting activities associated with orfamide A, which manifest themselves within minutes (Figs. 2c and 4c), this secondary metabolite also starts to disturb the morphology of *C*. *reinhardtii* over a time scale of 24 h (Fig. 5a). While staining with Evans blue indicates that orfamide A does not disrupt the algal cell membrane on the short term (Fig. 5b), membrane disruption may occur on the long term. Whether the different effects of orfamide A on *C*. *reinhardtii* are based on a single or multiple molecular interactions with components of the algal plasma membrane needs to be addressed in future work. In zoospores of the oomycete *Phytophthora ramorum*, a nonphotosynthetic member of the Heterokontophyta, orfamide A causes both immobilization and cell lysis within minutes^31^. The structure of the cell wall, which is absent from *P*. *ramorum* zoospores, and also the composition of the plasma membrane may explain why orfamide A exerts different effects on *P*. *ramorum* ^31^ and different photosynthetic algae (Table 1; Figs. 1d and 2c).

Mutation of *ofaA* in *P*. *protegens* results in the stop of orfamide production and defects in swarming (movement on soft agar) of the bacteria^31^. Bacterial lipopeptides, specifically orfamides, have been known to play different roles, e.g., as biosurfactants, as antifungal biocontrol agents, or as insecticides^31, 43^. We have now revealed an interkingdom function of orfamides A and B as precise saboteurs of eukaryotic signaling pathways, resulting in the immobilization of flagellated algal cells by interference with intracellular Ca^2+^ homeostasis. These cyclic lipopeptides thus represent novel biotic factors that cause Ca^2+^-dependent deflagellation in microalgae. Experiments with the Δ*ofaA* mutant (Fig. 4e, f, g) showed that orfamides are not essential for *P*. *protegens* to induce Ca^2+^ signaling and deflagellation in *C*. *reinhardtii*. The algal Ca^2+^ signal elicited by the Δ*ofaA* mutant was slightly faster than the signal elicited by the wild type (Fig. 4e), whereas deflagellation was delayed when an extract from the Δ*ofaA* mutant was used (Fig. 4f). Although the reason for these different effects is currently unclear, not every Ca^2+^ signal triggers deflagellation (as mentioned above), and different Ca^2+^ signatures may induce different downstream responses with different efficiencies. Our findings with the Δ*ofaA* mutant further show that *P*. *protegens* also produces other chemical mediators in addition to orfamides that possess Ca^2+^-eliciting and deflagellating activities, albeit rapid deflagellation (within 30 s) seems to be mainly associated with exposure to orfamides (Fig. 4f). Although these mediators may employ the same or alternative signaling pathways compared to orfamide A, the observed redundancy may indicate that Ca^2+^ elicitors and deflagellating compounds could aid to improve the fitness of *P*. *protegens*. In any case, the recovery of algal growth in the presence of the Δ*ofaA* mutant after ~10 days (Fig. 4g) indicates that a few algae survive the encounter when orfamides are absent, highlighting that orfamides help *P*. *protegens* to compete with *C*. *reinhardtii*.

**Fig. 6:**
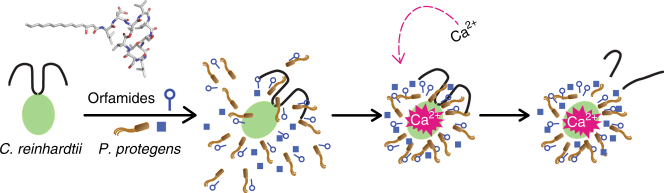
Proposed model of paralysis of algal cells by *P*. *protegens*. During the antagonistic interaction of *P*. *protegens* with *C*. *reinhardtii*, the cyclic lipopeptide orfamide A as well as yet unknown other chemical mediator(s) (indicated by blue squares) produced by the bacteria accumulate around the algal cells. Orfamides and the other compound(s) elicit a cytosolic Ca^2+^ signal in the green algae by triggering the influx of external Ca^2+^, at least in the case of orfamide A. Algal cells then become paralyzed by deflagellation, which seems to help *P*. *protegens* to acquire micronutrients

The strategy used by *P*. *protegens* (Fig. 6) differs markedly from previously reported tactics of bacteria that secrete algicidal agents and results in high concentrations of orfamides around the algal cells simply by the accumulation of the bacteria around the algae, as visualized in Supplementary Movie 1. This behavior results in the stop of the algal growth and altered cell morphology (Fig. 1b, d). We hypothesize that immobilization of the algal cells helps the bacteria to acquire nutrients from the algae. Bacterial growth is enhanced in coculture when the surrounding medium lacks trace elements (Supplementary Fig. 2), which are most likely delivered by the algal cells. However, the evidence for a benefit for the bacteria is currently limited, and further experiments are necessary to identify the specific nutrients obtained from the compromised algal cells. In addition to one or more trace elements, phosphate or sources of carbon or nitrogen that are present in the used medium may also have a role in the algal–bacterial interplay.

Beyond the new molecular insights, our findings also seem to be relevant from an ecological perspective because they contribute to a better mechanistic understanding of algal blooms that are part of phytoplankton. Algal blooms can affect the environment in two ways; they are not only major contributors to global photosynthesis, but can also be highly toxic, poisoning fish and shellfish^44^. Unicellular flagellated algae and colony-forming algae such as *Gonium* or *Pleodorina*, which are related to *C*. *reinhardtii*, can also form blooms^45^. Our data show that closely related wet soil and freshwater, as well as marine Chlorophyceae are likely subject to a similar deflagellation mechanism as *C*. *reinhardtii* because they get immobilized by orfamide A (Table 1). *P*. *protegens* is found in all these environments^17, 46^, and *P*. *protegens* and a closely related *P*. *fluorescens* strain have been reported to control algal blooms in freshwater and marine ecosystems, respectively^3, 44^. Therefore, the microbial interaction that we unraveled seems to be transferable to other flagellate Chlorophyceae algae, thus providing new tools to interfere with algal blooms of such species.

## Methods

### Strains and culture conditions

*C*. *reinhardtii* strain SAG 73.72 (mt^+^) was used as a wild type for most of the performed experiments. In some cases, mutant *adf-1* (CC-2919) with a defect TRP15 Ca^2+^ channel was used together with its parental strain CC-620. SAG 73.72 was obtained from the algal culture collection in Göttingen and CC-620, as well as the mutant CC-2919 from the Chlamydomonas Center at the University of Minnesota, USA. *C*. *reinhardtii* cells were grown in Tris-acetate-phosphate (TAP) medium^47^ in a light dark regime of 12:12 (unless otherwise indicated) with a light intensity of 50 µmol photons m^−2^ s^−1^ and stirring (250 rpm). The bacterial strains *F*. *johnsoniae* UW101^48^, *X*. *campestris* pv. *campestris* ATCC33913 ^49^, and *P*. *fluorescens* Pf-5^50^ renamed to *P*. *protegens* Pf-5^17^ were usually grown in LB medium at 28 °C with orbital shaking (200 rpm). The Δ*ofaA* deletion mutant^25^ was kindly provided by H. Gross (University of Tübingen), which he had obtained in turn from J.E. Loper (Oregon State University, USA) and B.T. Shaffer (OSU and UCSD-ARS). For the agar-based coculture assay, algal cells were grown under continuous light conditions (50 µmol photons m^−2^ s^−1^). Bacterial cells were grown in TAP medium for this coculture assay. Transgenic cell lines expressing the apo-aequorin reporter (AEQ, see below) were maintained on TAP agar plates (2% agar) with hygromycin B (20 µg ml^−1^). For Ca^2+^ measurement experiments, algal cells were grown in TAP medium without any antibiotics and the bacteria were grown in LB medium.

Other flagellate algae were obtained from the culture collection of algae and protozoa of the Scottish Marine Institute in United Kingdom (*Haematococcus pluvialis*, strain CCAP 34/8 and *Gonium pectorale*, strain CCAP 32/4), from the culture collection in Göttingen, Germany (*Chlamydomonas* sp., strain SAG 25.89 that was identified as CCMP 235, see Supplementary Fig. 6 and *Euglena gracilis*, strain SAG 1224-5/25) or were kindly provided by C. Wilhelm (*Pedinomonas minor*, SAG 1965-3), University of Leipzig, Germany. These flagellate algae were grown in media whose recipes are provided by the mentioned algal collections. Briefly, *H*. *pluvialis*, *G*. *pectorale*, and *P*. *minor* were grown in 3N-BBM + V medium, *E*. *gracilis* in 3N-BBM+V supplemented with 1 g l^−1^ sodium acetate trihydrate, 1 g l^−1^ meat extract, 2 g l^−1^ tryptone, and 2 g l^−1^ yeast extract, and *Chlamydomonas* sp. SAG 25.89 in a modified version of 3N-BBM+V where 32 g l^−1^ NaCl had been added and the NaNO_3_ had been replaced with 7 mM NH_4_Cl.

### Co-cultivation of *C*. *reinhardtii* and bacteria

For mixed cultivation of algae and bacteria on agar plates, a TAP plate (2% agar, 92 mm diameter) was overlaid with 3.3 ml 0.5% TAP agar (cover agar) containing 2 × 10^6^ *C*. *reinhardtii* cells. Overall, 15 µl of an overnight bacterial culture (containing between 2.1 and 2.5 × 10^6^ cells) were then applied to the surface of the solidified plate. The plates were incubated under continuous light (50 µmol photons m^−2^ s^−1^) at 20 °C and documented by scanning. For the mixed cultivation of algae and bacteria in liquid culture, *C*. *reinhardtii* and bacteria were grown in 100 ml conical flasks containing 50 ml TAP medium under continuous light (50 µmol photons m^−2^ s^−1^) and stirring (200 rpm) at 20 °C. Algal cell densities were determined by counting using an improved Neubauer chamber (no. T729.1, Carl Roth, Karlsruhe, Germany). Bacterial cell densities were determined as colony-forming units (CFU) by plating diluted bacterial culture on LB agar plates and counting CFUs.

For recording the cell densities of algae (*C*. *reinhardtii*) alone and in coculture with bacteria (*P*. *protegens* or the Δ*ofaA* mutant) (Supplementary Fig. 12), cells were grown in 50 ml NUNC cell culturing flasks containing 20 ml of TAP medium on a rotary shaker (200 rpm) at 23 °C. For each time-point, 1 ml of culture was transferred into a 2 ml Eppendorf tube, cooled down to 4 °C and centrifuged for 2.5 min at 200×*g*. The supernatant containing the bacteria was transferred to a new Eppendorf tube and the pellet bearing the algal cells was resuspended in 1 ml of TAP medium. OD was then measured at 700 nm and the cell density was estimated using a calibration curve done with the axenic cultures of *C*. *reinhardtii*.

For mixed cultivation of *C*. *reinhardtii* and *P*. *protegens* in liquid TAP medium free of trace elements (TAP-TE), bacteria were washed three times in TAP-TE medium and used to inoculate 50 ml TAP-TE in 100 ml conical flasks to an initial density of 10^6 ^cells ml^−1^. Bacteria were grown under continuous light (50 µmol photons m^−2^ s^−1^) and stirring (200 rpm) at 20 °C. After 24 h, algae were added at a ratio of ~1:100 algae to bacteria, and the growth of the bacteria was determined by plating different dilutions on LB agar plates and counting CFUs.

To record Supplementary Movie 1, wild-type cells of *C*. *reinhardtii* were grown to a cell density of 3–4 × 10^6^ cells ml^−1^. 20 µl of algal cells in TAP medium were dripped on a glass slide and a coverslip was placed gently over the spot. A total of 10 µl of a bacterial culture grown overnight (in LB medium) were introduced from the corner of the coverslip. The cells were observed under the microscope at ×400 magnification using the software Zen (blue edition, Carl Zeiss).

To record Supplementary Movies 2–15 (documentation of the effect of orfamide A on the motility of *C*. *reinhardtii* and other flagellate algae), 18 µl of algal culture (3 × 10^6^ ml^−1^) were placed as drop on an 8 well diagnostic glass slide (Thermo scientific) except for *E*. *gracilis*, where a concentration of 6 × 10^5^ ml^−1^ cells was used due to its large cell size. Overall, 2 µl of orfamide A in the corresponding medium were carefully added to the droplet to reach a final concentration of 35 µM orfamide A. The cells were visualized and recorded in movie using the software Zen (Blue edition, Carl Zeiss) before and after 30–60 s adding orfamide A. In case of *P*. *minor* and *E*. *gracilis*, movies were taken after 5 min to verify that orfamide A still has no effect after a longer period. All experiments for the Supplementary Movies were replicated twice.

### Acridine orange staining and fluorescence microscopy

*C*. *reinhardti* (4 × 10^6^ cells ml^−1^) and *P*. *protegens* (1.7 × 10^9^ CFU ml^−1^) cells were mixed in a ratio of algae to bacteria of ~1:400. 50 µl of the mixed cell suspension were applied on glass slides coated with poly-l-lysine. The suspension was allowed to stand for 10 min at room temperature. After incubation, the slide was dipped in ice cold methanol for 20 s followed by drying. Using a modified protocol^51^, the slide was stained with 100 µg ml^−1^ acridine orange in 1% (v/v) acetic acid for 2 min followed by gentle washing with deionized water. The cells were visualized using a fluorescence microscope (Axiophot, Carl Zeiss) equipped with suitable filters (excitation at 450–490 nm and emission at 520 nm). The images were recorded using Axiocam ICc1 camera along with the software Zen (blue edition) at a magnification of 1000 x.

### MALDI-imaging mass spectrometry

The main challenge for microbial MALDI-imaging mass spectrometry is to ensure complete contact between the sample and the conductive surface where the measurements take place^52^. While microbes can be cultured on non-disposable MALDI target slides^52^, we used indium tin oxide (ITO) coated glass slides that allowed storage of samples for several months and re-analysis if needed. Furthermore, our sample preparation method avoids transfer of delicate samples before MALDI-imaging mass spectrometry measurements, and it prevented flaking of the agar layer during the drying step, measurement, and storage.

For mixed cultivation of algae and bacteria, sterilized ITO coated glass slides (Bruker Daltonics) were placed inside petri dishes and overlaid with 3.3 ml 0.5% TAP agar containing *C*. *reinhardtii* at a density of ~9 × 10^5^ cells ml^−1^. 10 µl of *P*. *protegens* (~1 × 10^9^ cells ml^−1^) washed in TAP medium were then applied to the surface of the solidified agar medium. For comparison, axenic samples were prepared in the same way, but by omitting either algal or bacterial cells. The petri dishes were closed and wrapped with parafilm. The cultures were incubated at 20 °C and continuous light (50 µmol photons m^−2^ s^−1^). After 3 days, the parafilm and any condensed water were removed, and the petri dishes were wrapped with Micropore tape (3 M). In this way, the slides were stored at room temperature for additional 3 days until the agar was dried.

Matrix preparation followed the protocol proposed in Hoffman and Dorrestein, 2015^53^. Samples were coated with a saturated solution (20 mg ml^−1^) of universal MALDI matrix (1:1 mixture of 2,5-dihydroxybenzoic acid [DHB] and α-cyano-4-hydroxycinnamic acid [HCCA]; Sigma Aldrich) dissolved in ACN/MeOH/H_2_O (70:25:5, v/v/v). The matrix was sprayed using the automatic system ImagePrep device 2.0 (Bruker Daltonics) in 60 cycles (2 s spraying, 10 s incubation time, and 40 s of active drying using nitrogen gas), rotating the sample 180° after 30 cycles.

The samples were analyzed in an UltrafleXtreme MALDI TOF/TOF (Bruker Daltonics), which was operated in positive reflector mode using flexControl 3.0. The analysis was performed in two ranges: 0–2000 Da range, with 30% laser intensity (laser type 3), accumulating 1000 shots by taking 50 random shots at every raster position; and 700–3500 Da range, with 40% laser intensity (laser type 3), accumulating 1000 shots by taking 50 random shots at every raster position. Raster width was set at 200 µm. Calibration of the acquisition method was performed externally using Peptide Calibration Standard II (Bruker Daltonics) containing bradykinin 1–7, angiotensin II, angiotensin I, substance P, bombesin, ACTH clip 1-17, ACTH clip 18–39, and somatostatin 28.

In addition, spectra obtained in the 0–2000 Da range were processed with baseline subtraction in flexAnalysis 3.3 and corrected internally using the peaks of HCCA (from the matrix; [M+H]^+^ *m*/*z* 190.0499 and [2 M+H]^+^ *m*/*z* 379.0925). Processed spectra were uploaded in flexImaging 3.0 for visualization and SCILS Lab 2015b for analysis and representation. Chemical images were obtained using total ion count (TIC) normalization and weak denoising.

### Extraction of extracellular compounds from *P*. *protegens*

To prepare spent media extracts for LC-high-resolution MS measurements, starter cultures of *P*. *protegens* were grown in 5 ml LB broth in test tubes for 24 h at 28 °C and orbital shaking at 200 rpm. ~7 × 10^8^ cells from 0.5 ml of starter culture were washed three times with TAP medium and used to inoculate 250 ml of TAP medium in a 1 liter conical flask. The culture was grown at room temperature with shaking at 110 rpm. To prepare cocultures, *C*. *reinhardtii* cells were added after 24 h in a ratio of 1:100 (algae to bacteria) and incubated for further 24 h. The cells were removed by centrifugation (20 min, 16,000×*g*, 4 °C). The supernatant was sterile filtrated (CHROMAFIL® CA-20/25 (S), pore size 0.20 µm), extracted three times with ethyl acetate (250 ml), and the extract was dried by rotary evaporation at 40 °C. The dried residue was dissolved in 250 µl of methanol (HPLC grade), and the solution passed through a filter with a pore size of 0.45 µm.

### LC-high-resolution MS

The analyses were performed on a Q-Exactive Orbitrap mass spectrometer coupled to an Accela AS LC system (Thermo Fisher, San José, USA). Organic solvents (LC-MS grade) and reagents used for LC-MS analysis were purchased from Sigma Aldrich. An Accucore C18 column (2.6 µm, 100 × 2.1 mm; Thermo Fisher) was eluted at 0.2 ml min^−1^ with water (eluent A) and acetonitrile (eluent B) both containing 0.1% formic acid. Gradient elution used was: 5–98% B over 10 min, hold 12 min, 98–5% B in 0.1 min, and hold 6.9 min. Injection volume was 10 µl and the oven temperature was 30 °C. Pure orfamide A and B (Santa Cruz Biotechnology) was dissolved in methanol/water (1:1, *v*/*v*) to a concentration of 100 ng ml^−1^. High-resolution full MS experiments (positive ionization) were acquired in the range of *m*/*z* 150–2000. The following source settings were used: spray voltage = 3.5; capillary temperature = 250 °C; capillary voltage = 50 V; sheath gas flow = 49 and auxiliary gas flow = 5 (arbitrary units); tube lens voltage = 130 V. Resolving power was set at 35,000 (FWHM at *m*/*z* 400). For fragmentation experiments, precursors were selected by the quadrupole with an isolation window of 1 Da, a target value of 2 × 10^5^, and a maximum ion injection time of 200 ms. Precursor ions were subjected to fragmentation with 18 and 22% normalized collision energy in the higher energy collisional dissociation (HCD) cell, and the resulting fragment ions were detected in the Orbitrap mass analyzer at a resolution of 70,000 (FWHM at *m*/*z* 400).

### Aequorin expression and transformation of *C*. *reinhardtii*

The pHD-AEQ2 vector was used for transformation. Its complete sequence is shown in Supplementary Fig. 7. This vector carries a codon optimized *apo-aequorin* gene^27^ that was synthesized by GeneArt Life Technologies. The *apo-aequorin* gene was put under control of the *HSP70A*/*RBCS2* tandem promoter along with the first intron of *RBCS2* ^54^ and was set in front of the *RACK1* 3′-UTR representing a constitutive reference transcript^55^. The vector also carries the hygromycin B resistance from the Hyg3 vector^56^ for selection in *C*. *reinhardtii* as well as an ampicillin resistance (selection marker for *E*. *coli*). *C*. *reinhardtii* wild-type cells (strain SAG 73.72) were transformed with the autolysin method as described earlier^57^. SAG 73.72 cells were transformed with either pHyg3 (vector control) or pHD-AEQ2 (AEQ lines). Hygromycin-resistant colonies were further examined in immunoblots (see below) for expression of apo-aequorin.

### Crude extract and immunoblotting

Preparation of crude extracts to detect the apo-aequorin protein was done as described^57^. For immunoblotting, 50 µg of proteins of a crude extract from cells harvested in the middle of the day were separated by 10% SDS-PAGE and the proteins were transferred to a polyvinylidine difluoride (PVDF) membrane. Blocking and detection was done as published before^58^. The primary antibody used for immunoblotting was anti-aequorin (Covalab, France); it was used at a dilution of 1:2000 in the blocking solution. The secondary antibody was anti-rabbit antibody conjugated to horseradish peroxidase used at a dilution of 1:6666 (Sigma Aldrich). Full blots and stained membranes are presented in Supplementary Fig. 14.

### Intracellular Ca^2+^ measurement using an aequorin reporter

Cells expressing apo-aequorin (SAG 73.72::pHD-AEQ2, abbreviated as AEQ cells) as well as the control cells used for the Ca^2+^ measurement were grown in TAP medium for 72 h up to a cell density of 3–4 × 10^6^ cells ml^−1^. The cells were counted using a Thoma cell counting chamber. Prior to the Ca^2+^ measurement, 1 ml of cells were incubated together with 23 µM of coelenterazine (Synchem, Germany) in the dark for 2 h at room temperature. For the measurement, 96 well plates (white with clear bottom, Berthold technologies #60705) were used. Each well was loaded with 100 µl of coelenterazine-treated cell suspension. The microplate luminometer (LB-941, Mithras Berthold Technologies) was used for the luminescence measurement. The machine was programmed using the software Mikrowin2000 (provided by the manufacturer). The counting time was set between 0.2 and 1 s and the delay between each count was set between 5 and 7 s. For measuring the Ca^2+^ elevation under the influence of 20 mM CaCl_2_, 190 µl of cells (AEQ22, AEQ24, AEQ33, or AEQ34) incubated with coelenterazine were loaded in a 96 well microplate; after measurement of background, 10 µl of 400 mM CaCl_2_ solution was injected followed by RLU measurement for 300 s. Maximal luminescence (full discharge) was determined by adding 100 µl of discharge solution (2 M CaCl_2_, 10% ethanol) to each well^59^. Obtained relative luminescence unit (RLU) values were exported to Microsoft Excel and values were converted to molar Ca^2+^ concentrations using the equation pCa = 0.332588(–log *k*) + 5.5593 as in ref.^59^.

### Induction of pH and salt stress

Acid-induced deflagellation was done as described^26^. 100 µl of 40 mM sodium acetate, pH 4.5 (40 mM sodium acetate, pH adjusted using acetic acid) were added to 100 µl coelenterazine pretreated AEQ34 cells in HEPES buffer (10 mM HEPES, pH 7.4, 1 mM CaCl_2_, 50 µM MgCl_2_, and 1 mM KCl). For salt stress measurements, 100 µl of NaCl (in ddH_2_O) were added to 100 µl of AEQ34 cells to reach a final concentration of 100, 200, or 300 mM.

### Ca^2+^ measurement under the influence of orfamide A and B

Algal cells were grown in TAP medium for 72 h, the cell density was adjusted to 4 × 10^6^ cells ml^−1^, and then the cells were pre-incubated with coelenterazine. *P*. *protegens* was grown in LB medium overnight. Orfamide A or B (Santa Cruz Biotechnology) were dissolved in methanol. For the Ca^2+^ measurement, this stock solution was diluted in 100 µl of TAP medium before commencing the measurement. To block the Ca^2+^ channels in the plasma membrane, AEQ34 cells grown in TAP medium were suspended in HEPES buffer (see above) after washing the cells three times with the same. Overall, 20 µM lanthanum chloride (LaCl_3_) was added to the cells before starting the experiment in the 96 well microplate. For preparing TAP medium lacking Ca^2+^, the salt solution mix for TAP deficient of calcium chloride was used.

### Deflagellation and Evans blue staining

Cells were taken at a cell density of 3–4 × 10^6 ^cells ml^−1^. For Evans blue staining, the cells were treated with 10 or 35 µM of orfamide A, 10 µM orfamide B, or 10 µM of mastoparan (Sigma Aldrich, product number M5280) for 30 s followed by brief washing three times for 10 s with TAP medium at room temperature. As control, the samples were treated with an equivalent volume of methanol for orfamides and water in the case of mastoparan. After washing the cells, they were resuspended in 0.1% (w/v) Evans blue stain in TAP medium for 5 min followed by microscopy and cell counting. For deflagellation studies, the cells were treated in similar manner mentioned above, but the washing steps were skipped as the cells were fixed with 4–8% (v/v) potassium iodide after treatment with orfamide and mastoparan. The cells were imaged using DIC microscopy (Axioplan 2 from Zeiss), and counting was performed using Image J (Version 1.48) cell counter.

### Data availability

The datasets generated during and/or analyzed during the current study are available from the corresponding authors on reasonable request. Plasmids and transgenic lines are available from MM on basis of a Material Transfer Agreement.

## Electronic supplementary material

Supplementary Information

Description of Additional Supplementary Files

Supplementary Movie 1

Supplementary Movie 2

Supplementary Movie 3

Supplementary Movie 4

Supplementary Movie 5

Supplementary Movie 6

Supplementary Movie 7

Supplementary Movie 8

Supplementary Movie 9

Supplementary Movie 10

Supplementary Movie 11

Supplementary Movie 12

Supplementary Movie 13

Supplementary Movie 14

Supplementary Movie 15
