# Supplementary material for: Antagonistic bacteria disrupt calcium homeostasis and immobilize algal cells
Source: Nat Commun. 2017 Nov 24;8:1756. doi: 10.1038/s41467-017-01547-8 (PMC5701020; doi:10.1038/s41467-017-01547-8)
Supplement: Supplementary file 1 — Supplementary Information [file 41467_2017_1547_MOESM1_ESM.pdf]

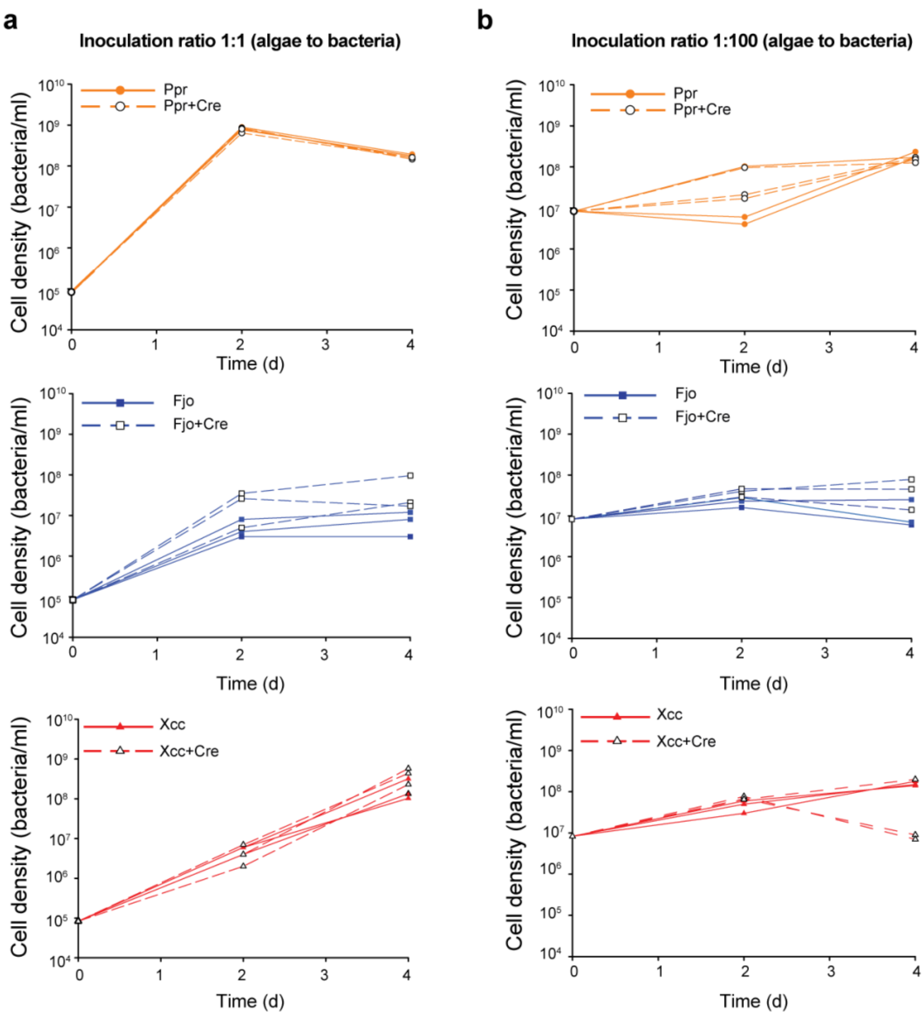

Supplementary Figure 1 | Mixed cultivation of *C. reinhardtii* with different heterotrophic bacteria showing similar growth rates of the bacteria in pure and mixed cultures. (a) Liquid cocultivation using a 1:1 ratio of algae to bacteria for inoculation. Cultures were inoculated to obtain initial cell densities of 8.3 x 10^4 algae ml^{-1} and in coculture 8.3 x 10^4 bacteria ml^{-1} were added. Bacterial cell densities were determined by counting colony-forming units on LB agar plates. Values of triplicate cultures are shown. **(b)** Liquid cocultivation using a 1:100 ratio of algae to bacteria for inoculation. Cultures were inoculated to obtain initial cell densities of 8.3 x 10^4 algae ml^{-1} and in coculture 8.3 x 10^6 bacteria ml^{-1} were added. Otherwise, set-up and analysis were done as in (a). Abbreviations: Cre, *Chlamydomonas reinhardtii*; Fjo, *Flavobacterium johnsoniae*; Xcc, *Xanthomonas campestris* pv. *campestris*; Ppr, *Pseudomonas protegens*; dpi,

days post inoculation. In (a)-(b), all experiments were performed once with three biological replicates.

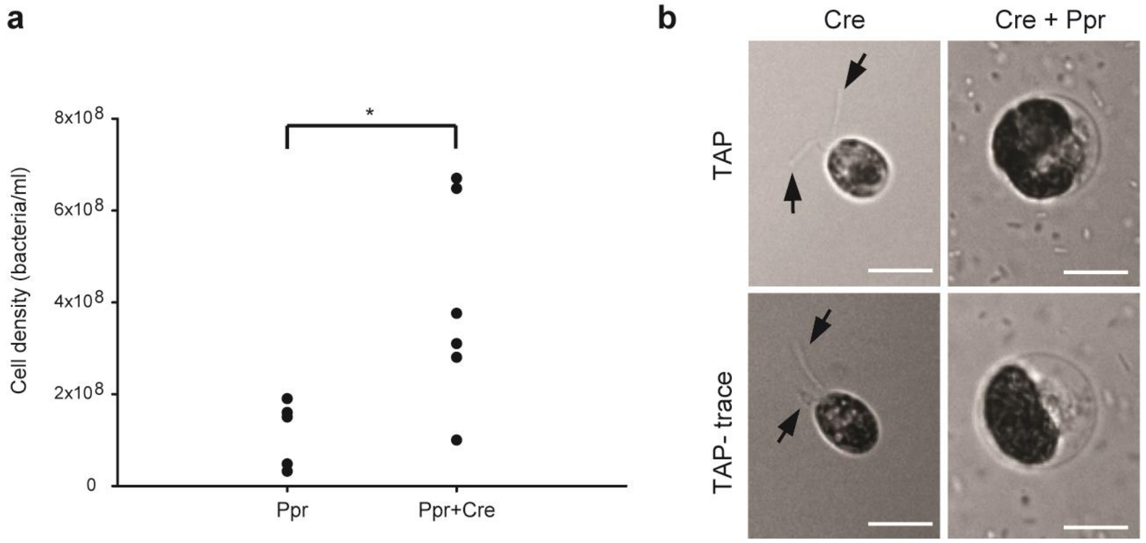

Supplementary Figure 2 | Evidence that *P. protegens* can acquire micronutrients from *C. reinhardtii* under nutrient-limiting conditions. (a) Cell density of *P. protegens* (Ppr) in absence or presence of *C. reinhardtii* (Cre) in medium without micronutrients. Washed bacterial cells were grown for 24 h in liquid TAP medium free of trace elements[1]. Then *C. reinhardtii* was added (~1:100 ratio of algae to bacteria) and grown for further 24 h (Ppr+Cre) or the bacteria were grown alone for further 24 h (Ppr). Under these nutrient-limiting conditions, there was no significant growth of the algae over the measured period. The asterisk indicates a statistically significant difference ($p < 0.05$) according to the two-tailed approximate t-test[2]. This test was used because the variances of the two groups are significantly different according to the two-tailed Snedecor's F-test. The result of a Shapiro-Wilk test (http://contchart.com/goodness-of-fit.aspx) was in agreement with normally distributed populations. This experiment was performed twice with three biological replicates each (n = 6 for Ppr + Cre, n = 5 for Ppr; one replicate was discarded due to technical problems). **(b)** Morphology of algal cells after 24 h of cocultivation with *P. protegens* in medium with (TAP) and without (TAP-trace) micronutrients. Algal cells

were fixed with 8% potassium iodide and visualized using bright field microscopy at 400x magnification (scale bar: 10 µm). The experiment was performed twice. Representative pictures of a single experiment are shown.

a

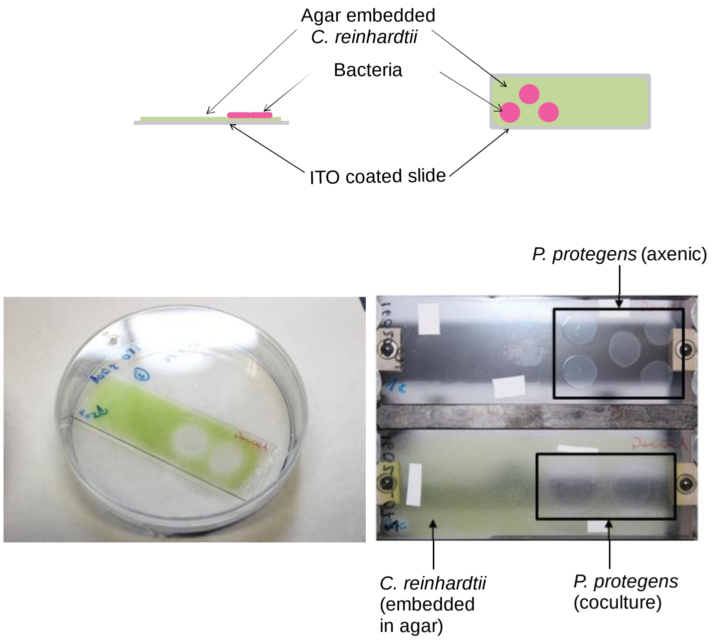

b

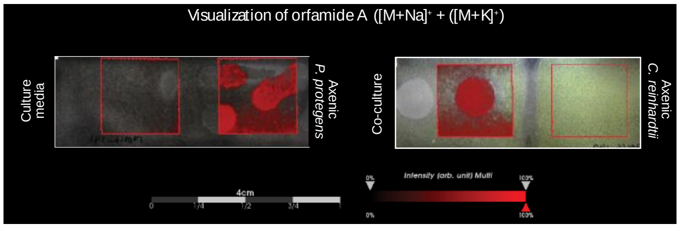

Supplementary Figure 3 | MALDI-imaging mass spectrometry (MALDI-IMS) of orfamide A in mixed cultures of *C. reinhardtii* and *P. protegens* and axenic cultures. (a) Algal cells (*C. reinhardtii*) were mixed with liquid agar medium and applied onto the surface of indium tin oxide (ITO)-coated glass slides (scheme at the top). Droplets of bacterial suspension were then applied to the top of the solidified agar. After growth for three days at 20 °C and continuous light (50 µmol photons m^{-2} s^{-1}), the samples were dried, sprayed with MALDI matrix and analyzed. The photograph on the left shows the slide after drying, and the photograph on the right shows the mounted slides immediately before data acquisition. The white rectangles are reference points

used to align the optical image and for the analysis. Even after repeated measurements by MALDI-IMS, this sample preparation method completely prevented flaking of the agar layer, which has been described as the primary limitation of microbial IMS[3]. (**b)** MALDI-IMS reveals that *P. protegens* secretes orfamides. Visualization of orfamide A (*m/z* 1317.7 ($[M+Na]^+$) and *m/z* 1333.8 ($[M+K]^+$)) in cultures of axenic *P. protegens* and in coculture with *C. reinhardtii* by MALDI-TOF imaging mass spectrometry (see also Supplementary Fig. 4). The red squares delimit the measured area. The experiments were performed in three biological replicates. Three independent MALDI-mass spectrometry imaging experiments were conducted.

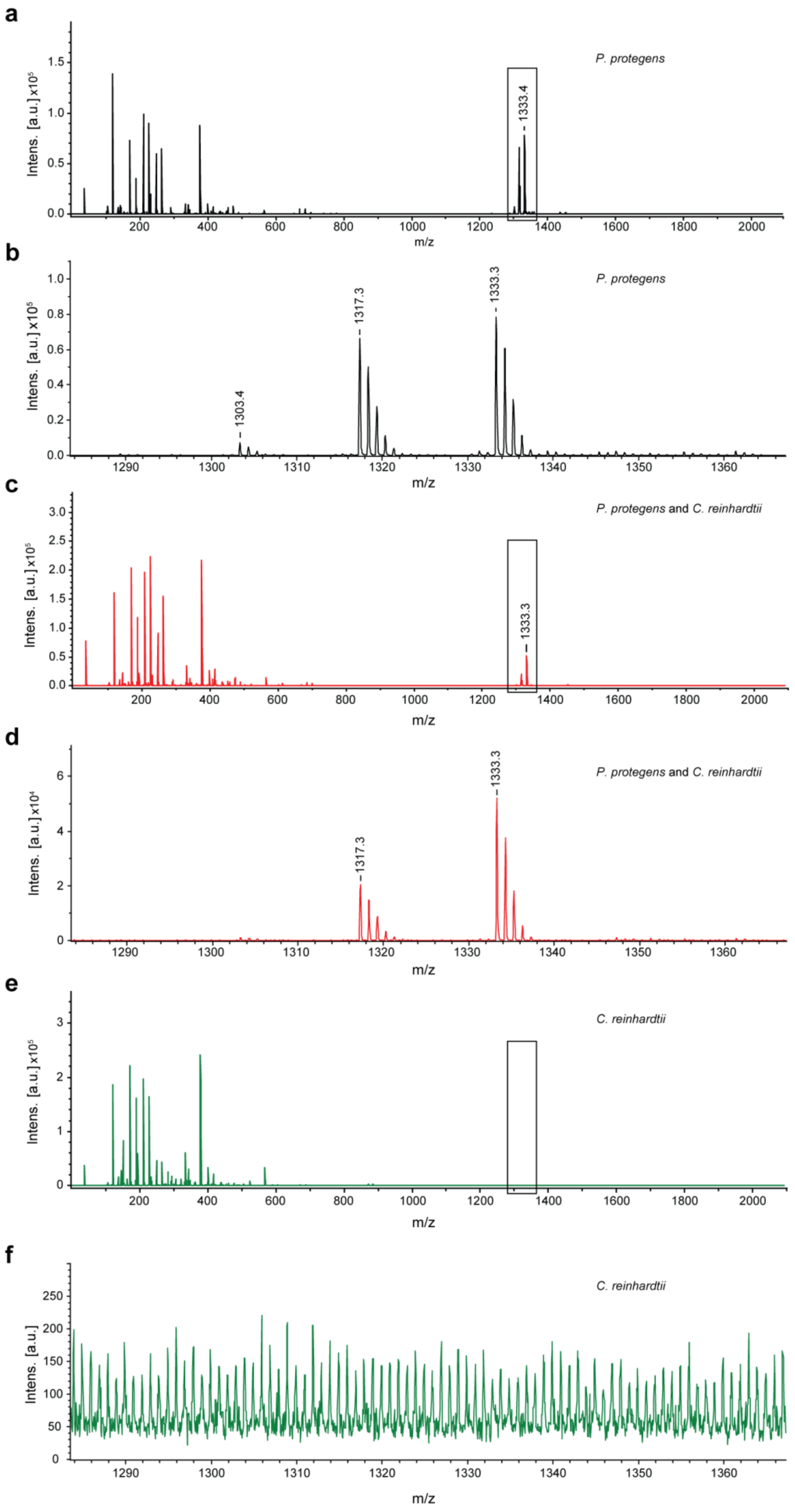

a
P. protegens
1333.4
Intens. [a.u.] x10⁵
m/z
b
P. protegens
1303.4
1317.3
1333.3
Intens. [a.u.] x10⁵
m/z
c
P. protegens and C. reinhardtii
1333.3
Intens. [a.u.] x10⁵
m/z
d
P. protegens and C. reinhardtii
1317.3
1333.3
Intens. [a.u.] x10⁴
m/z
e
C. reinhardtii
Intens. [a.u.] x10⁵
m/z
f
C. reinhardtii
Intens. [a.u.]
m/z

Supplementary Figure 4 | Individual MALDI mass spectra from different regions of the Imaging MS experiment. (a) Mass spectrum from axenic *P. protegens*. **(b)** Enlarged spectrum of the boxed region from (a) containing the peaks of orfamides. **(c)** Mass spectrum from *P. protegens-C. reinhardtii* co-culture. **(d)** Enlarged spectrum of the boxed region from (c) containing the peaks of orfamides. **(e)** Mass spectrum from axenic *C. reinhardtii.* **(f)** Enlarged spectrum of the boxed region from (e). In (b) and (d), the most intense monoisotopic peaks are *m/z* 1317.3 and *m/z* 1333.3, corresponding to Na^+ and K^+ adducts of orfamide A ($C_{64}H_{114}N_{10}O_{17}$; exact mass 1294.8363 Da). In (b), *m/z* 1303.4 corresponds to the Na^+ adduct of orfamide B ($C_{63}H_{112}N_{10}O_{17}$; exact mass 1280.8207 Da). MALDI-imaging mass spectrometry experiments had three biological and one technical replicate, and they were performed three times.

a

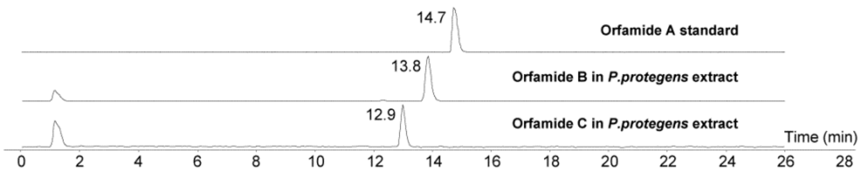

b

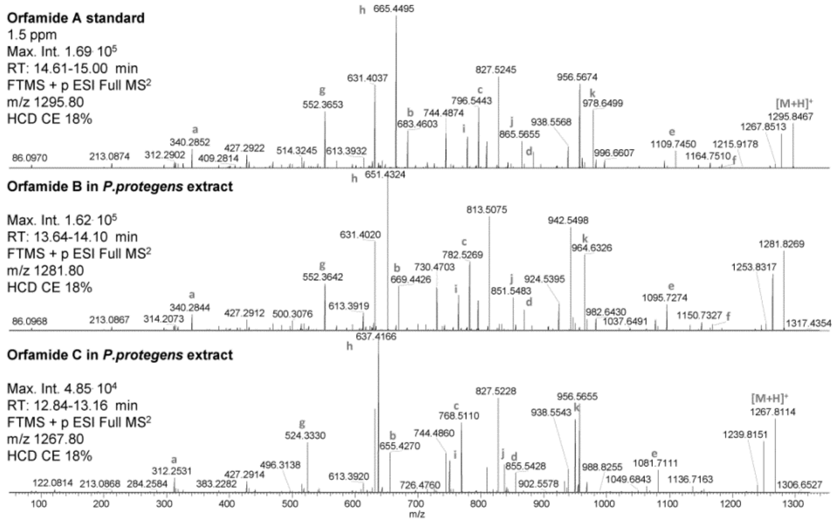

c

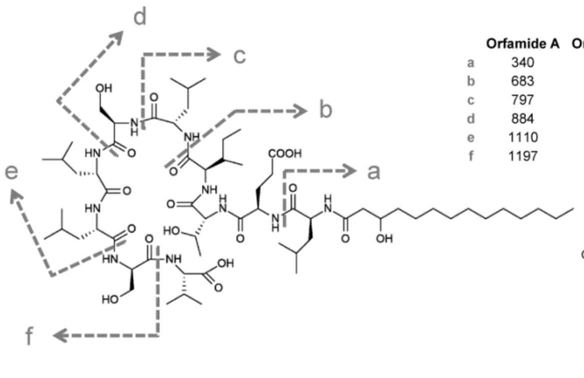

	Orfamide A	Orfamide B[1]	Orfamide C[2]
a	340	340	312
b	683	669	655
c	797	783	769
d	884	870	856
e	1110	1096	1082
f	1197	1183	1169

Orfamide A (cleaved at the ester bond)

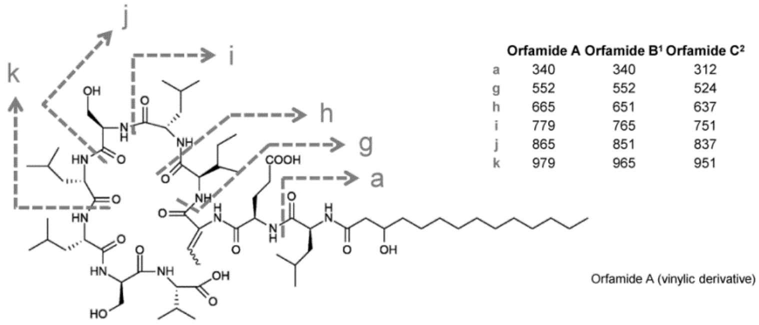

	Orfamide A	Orfamide B[1]	Orfamide C[2]
a	340	340	312
g	552	552	524
h	665	651	637
i	779	765	751
j	865	851	837
k	979	965	951

Orfamide A (vinylic derivative)

[1] Orfamide B: 7-Valine analogue
[2] Orfamide C: 10-N-(3-hydroxydodecanoyl)

Supplementary Figure 5 | Confirmation of orfamides B and C in *P. protegens* extract by LC-ESI-HRMS/MS. **(a)** Total ion chromatograms of an MS^2 analysis of orfamide A (from commercial standard, $C_{64}H_{114}N_{10}O_{17}$ accurate mass 1294.836345 u) at *m/z* 1295.8, and orfamide B ($C_{63}H_{112}N_{10}O_{17}$ accurate mass 1280.820695 u) and orfamide C ($C_{62}H_{110}N_{10}O_{17}$ accurate mass 1266.805045 u) from *P. protegens* extract at *m/z* 1281.8 and *m/z* 1267.8 respectively. **(b)** Full MS^2 scan spectra of orfamide A (from commercial standard), and orfamide B and C from *P. protegens* extract. Identified fragments are annotated with gray letters, following the name system in (c). In (a) and (b), the extract was prepared from the supernatant of an axenic *P. protegens* culture. **(c)** Fragmentation of orfamide A according to Gross et al. (2007)[4]. Orfamide A is cleaved at the ester bond, and the resulting linearized peptide fragments are shown in the first diagram. The vinylic derivative after water elimination from threonine fragments is shown in the second diagram. The tables show the calculated fragments for orfamide B (an isoleucine at the fourth position instead of a valine) and orfamide C (3-hydroxydodecanoic fatty acid chain instead of 3-hydroxytetradecanoic fatty acid chain).

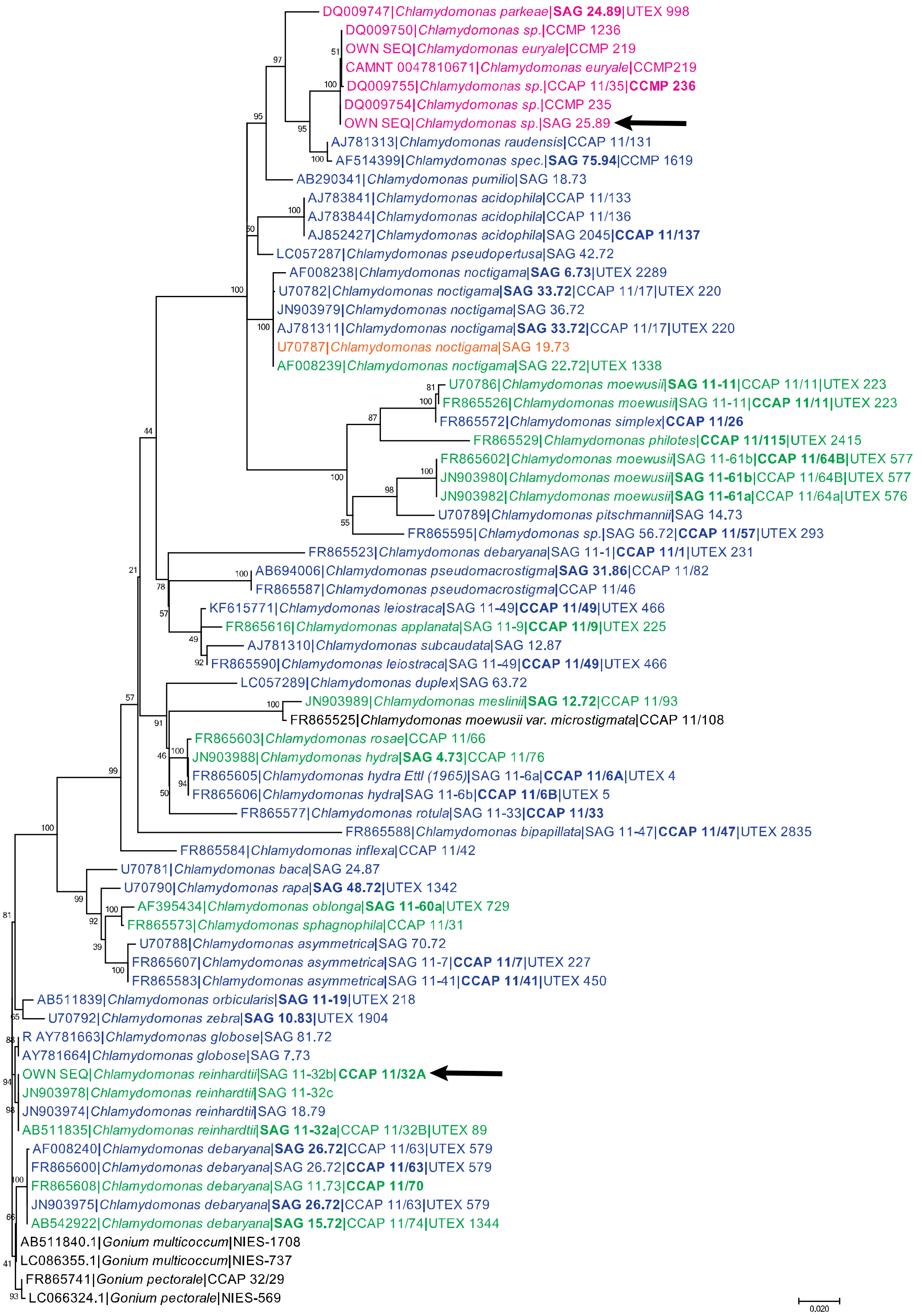
DQ009747|Chlamydomonas parkeae|SAG 24.89|UTEX 998
DQ009750|Chlamydomonas sp.|CCMP 1236
OWN SEQ|Chlamydomonas euryale|CCMP 219
CAMNT 0047810671|Chlamydomonas euryale|CCMP219
DQ009755|Chlamydomonas sp.|CCAP 11/35|CCMP 236
DQ009754|Chlamydomonas sp.|CCMP 235
OWN SEQ|Chlamydomonas sp.|SAG 25.89
AJ781313|Chlamydomonas raudensis|CCAP 11/131
AF514399|Chlamydomonas spec.|SAG 75.94|CCMP 1619
AB290341|Chlamydomonas pumilio|SAG 18.73
AJ783841|Chlamydomonas acidophila|CCAP 11/133
AJ783844|Chlamydomonas acidophila|CCAP 11/136
AJ852427|Chlamydomonas acidophila|SAG 2045|CCAP 11/137
LC057287|Chlamydomonas pseudopertusa|SAG 42.72
AF008238|Chlamydomonas noctigama|SAG 6.73|UTEX 2289
U70782|Chlamydomonas noctigama|SAG 33.72|CCAP 11/17|UTEX 220
JN903979|Chlamydomonas noctigama|SAG 36.72
AJ781311|Chlamydomonas noctigama|SAG 33.72|CCAP 11/17|UTEX 220
U70787|Chlamydomonas noctigama|SAG 19.73
AF008239|Chlamydomonas noctigama|SAG 22.72|UTEX 1338
U70786|Chlamydomonas moewusii|SAG 11-11|CCAP 11/11|UTEX 223
FR865526|Chlamydomonas moewusii|SAG 11-11|CCAP 11/11|UTEX 223
FR865572|Chlamydomonas simplex|CCAP 11/26
FR865529|Chlamydomonas philotes|CCAP 11/115|UTEX 2415
FR865602|Chlamydomonas moewusii|SAG 11-61b|CCAP 11/64B|UTEX 577
JN903980|Chlamydomonas moewusii|SAG 11-61b|CCAP 11/64B|UTEX 577
JN903982|Chlamydomonas moewusii|SAG 11-61a|CCAP 11/64a|UTEX 576
U70789|Chlamydomonas pitschmannii|SAG 14.73
FR865595|Chlamydomonas sp.|SAG 56.72|CCAP 11/57|UTEX 293
FR865523|Chlamydomonas debaryana|SAG 11-1|CCAP 11/1|UTEX 231
AB694006|Chlamydomonas pseudomacrostigma|SAG 31.86|CCAP 11/82
FR865587|Chlamydomonas pseudomacrostigma|CCAP 11/46
KF615771|Chlamydomonas leiostraca|SAG 11-49|CCAP 11/49|UTEX 466
FR865616|Chlamydomonas applanata|SAG 11-9|CCAP 11/9|UTEX 225
AJ781310|Chlamydomonas subcaudata|SAG 12.87
FR865590|Chlamydomonas leiostraca|SAG 11-49|CCAP 11/49|UTEX 466
LC057289|Chlamydomonas duplex|SAG 63.72
JN903989|Chlamydomonas meslinii|SAG 12.72|CCAP 11/93
FR865525|Chlamydomonas moewusii var. microstigmata|CCAP 11/108
FR865603|Chlamydomonas rosae|CCAP 11/66
JN903988|Chlamydomonas hydra|SAG 4.73|CCAP 11/76
FR865605|Chlamydomonas hydra Ettl (1965)|SAG 11-6a|CCAP 11/6A|UTEX 4
FR865606|Chlamydomonas hydra|SAG 11-6b|CCAP 11/6B|UTEX 5
FR865577|Chlamydomonas rotula|SAG 11-33|CCAP 11/33
FR865588|Chlamydomonas bipapillata|SAG 11-47|CCAP 11/47|UTEX 2835
FR865584|Chlamydomonas inflexa|CCAP 11/42
U70781|Chlamydomonas baca|SAG 24.87
U70790|Chlamydomonas rapa|SAG 48.72|UTEX 1342
AF395434|Chlamydomonas oblonga|SAG 11-60a|UTEX 729
FR865573|Chlamydomonas sphagnophila|CCAP 11/31
U70788|Chlamydomonas asymmetrica|SAG 70.72
FR865607|Chlamydomonas asymmetrica|SAG 11-7|CCAP 11/7|UTEX 227
FR865583|Chlamydomonas asymmetrica|SAG 11-41|CCAP 11/41|UTEX 450
AB511839|Chlamydomonas orbicularis|SAG 11-19|UTEX 218
U70792|Chlamydomonas zebra|SAG 10.83|UTEX 1904
R AY781663|Chlamydomonas globose|SAG 81.72
AY781664|Chlamydomonas globose|SAG 7.73
OWN SEQ|Chlamydomonas reinhardtii|SAG 11-32b|CCAP 11/32A
JN903978|Chlamydomonas reinhardtii|SAG 11-32c
JN903974|Chlamydomonas reinhardtii|SAG 18.79
AB511835|Chlamydomonas reinhardtii|SAG 11-32a|CCAP 11/32B|UTEX 89
AF008240|Chlamydomonas debaryana|SAG 26.72|CCAP 11/63|UTEX 579
FR865600|Chlamydomonas debaryana|SAG 26.72|CCAP 11/63|UTEX 579
FR865608|Chlamydomonas debaryana|SAG 11.73|CCAP 11/70
JN903975|Chlamydomonas debaryana|SAG 26.72|CCAP 11/63|UTEX 579
AB542922|Chlamydomonas debaryana|SAG 15.72|CCAP 11/74|UTEX 1344
AB511840.1|Gonium multicoccum|NIES-1708
LC086355.1|Gonium multicoccum|NIES-737
FR865741|Gonium pectorale|CCAP 32/29
LC066324.1|Gonium pectorale|NIES-569
0.020

Supplementary Figure 6 | Molecular phylogenetic analysis of *Chlamydomonas* species based on the *18S RNA* gene. Evolutionary analyses were conducted in MEGA7 (5). The evolutionary history was inferred by using the maximum likelihood method based on the general time reversible model. Bootstrap analysis was performed with 1000 replicates. The tree with the highest log likelihood (-8577.2167) is shown. A discrete gamma distribution was used to model evolutionary rate differences among sites (5 categories (+G, parameter = 0.2065)). The rate variation model allowed for some sites to be evolutionarily invariable ([+I], 36.7754% sites). The tree is drawn to scale, with branch lengths measured in the number of substitutions per site. The percentage of trees, in which the associated taxa clustered together, is shown next to the branches. The analysis involved 70 nucleotide sequences of the *18S RNA* gene, from which four *Gonium* spp. sequences were included as outgroup. There were a total of 1656 positions in the final dataset. The species have been colored according to the isolation habitat as specified by the algal culture collections: soil (green), freshwater (blue), brackish water (orange) or seawater (red, marked in magenta). Species in black have an unknown isolation habitat. For species present in different culture collections, the strain donor for DNA sequencing is marked in bold. The positions of *C. reinhardtii* and *Chlamydomonas* sp. SAG 25.89 are indicated with an arrow.

CTGACGCGCCCTGTAGCGGCGCATTAAGCGCGGCGGGTGTGGTGGTTACG

CGCAGCGTGACCGCTACACTTGCCAGCGCCCTAGCGCCCGCTCCTTTCGC

TTTCTTCCCTTCCTTTCTCGCCACGTTCGCCGGCTTTCCCCGTCAAGCTC

TAAATCGGGGGCTCCCTTTAGGGTTCCGATTTAGTGCTTTACGGCACCTC

GACCCCAAAAAACTTGATTAGGGTGATGGTTCACGTAGTGGGCCATCGCC

CTGATAGACGGTTTTTCGCCCTTTGACGTTGGAGTCCACGTTCTTTAATA

GTGGACTCTTGTTCCAAACTGGAACAACACTCAACCCTATCTCGGTCTAT

TCTTTTGATTTATAAGGGATTTTGCCGATTTCGGCCTATTGGTTAAAAAA

TGAGCTGATTTAACAAAAATTTAACGCGAATTTTAACAAAATATTAACGC

TTACAATTTCCATTCGCCATTCAGGCTGCGCAACTGTTGGGAAGGGCGAT

CGGTGCGGGCCTCTTCGCTATTACGCCAGCTGGCGAAAGGGGGATGTGCT

GCAAGGCGATTAAGTTGGGTAACGCCAGGGTTTTCCCAGTCACGACGTTG

TAAAACGACGGCCAGTGAGCGCGCGTAATACGACTCACTATAGGGCGAAT

TGGAGC**TCGCTGAGGCTTGACATGATTGGTGCGTATGTTTGTATGAAGCT**

ACAGGACTGATTTGGCGGGCTATGAGGGCGGGGGAAGCTCTGGAAGGGCC

GCGATGGGGCGCGCGGCGTCCAGAAGGCGCCATACGGCCCGCTGGCGGCA

CCCATCCGGTATAAAAGCCCGCGACCCCGAACGGTGACCTCCACTTTCAG

CGACAAACGAGCACTTATACATACGCGACTATTCTGCCGCTATACATAAC

CACTCAGCTAGCTTAAGATCCCATCAAGCTTGCATGCCGGGCGCGCCAGA

AGGAGCGCAGCCAAACCAGGATGATGTTTGATGGGGTATTTGAGCACTTG

CAACCCTTATCCGGAAGCCCCCTGGCCCACAAAGGCTAGGCGCCAATGCA

AGCAGTTCGCATGCAGCCCCTGGAGCGGTGCCCTCCTGATAAACCGGCCA

GGGGGCCTATGTTCTTTACTTTTTACAAGAGAAGTCACTCAACATCTTA

AAATGGCCAGGTGAGTCGACGAGCAAGCCCGGCGGATCAGGCAGCGTGCT

TGCAGATTTGACTTGCAACGCCCGCATTGTGTCGACGAAGGCTTTTGGCT

CCTCTGTCGCTGTCTCAAGCAGCATCTAACCCTGCGTCGCCGTTTCCATT

TGCAGG<u>ATGCTCGACGTGAAGCTGACCAGCGACTTCGACAACCCCCGCTG</u>

GATCGGCCGCCACAAGCACATGTTCAACTTCCTGGACGTGAACCACAACG

GCAAGATCAGCCTGGACGAGATGGTGTACAAGGCCAGCGACATCGTGATC

AACAACCTGGGCGCCACCCCCGAGCAGGCCAAGCGCCACAAGGACGCCGT

GGAGGCCTTCTTCGGCGGCGCCGGCATGAAGTACGGCGTGGAGACCGACT

GGCCCGCCTACATCGAGGGCTGGAAGAAGCTGGCCACCGACGAGCTGGAG

AAGTACGCCAAGAACGAGCCCACCCTGATCCGCATCTGGGGCGACGCCCT

GTTCGACATCGTGGACAAGGACCAGAACGGCGCCATCACCCTGGACGAGT

GGAAGGCCTACACCAAGGCCGCCGGCATCATCCAGAGCAGCGAGGACTGC

GAGGAGACCTTCCGCGTGTGCGACATCGACGAGAGCGGCCAGCTGGACGT

GGACGAGATGACCCGCCAGCACCTGGGCTTCTGGTACACCATGGACCCCG

CCTGCGAGAAGCTGTACGGCGGCGCGGTGCCCTAATCTAGA*ATGCCCAGC*

CGCGCCTAGCTGCGGCCTGGCCCTGCGGGCCAGGAGCATCGGGAGCTGGA

GGGGTTTAGGAGGAGGCGCGCAGCGGCGGCGGCTGCGCCACCTGTTGAGA

GGCGGGCGGCGTGCTGCGTCGCGGTGTGTTGAGGATGCGTCGCGGGGCTG

CCGGACTGGCGATCTGTAACGCGTTGATGCTGGTCAGTCTCATTTCACGT

*GTTCTCCCACGTTCTCTCTTGCGGAATGTAAGGTGTGCGTGAT*GGCGGCT

ACCAAGGGAGGCCTAGGGAACTTGGAAAGGAACTCCCGGGTAAGTAGCTG

AGAAGAGGCCCGCACCGATCGCCCTTCCCAACAGTTGCGCAGCCTGAATG

GCGAATGGCGCCTGATGCGGTATTTTCTCCTTAGATATCAAGCTTCTTTC

TTGCGCTATGACACTTCCAGCAAAAGGTAGGGCGGGCTGCGAGACGGCTT

CCCGGCGCTGCATGCAACACCGATGATGCTTCGACCCCCCGAAGCTCCTT

CGGGGCTGCATGGGCGCTCCGATGCCGCTCCAGGGCGAGCGCTGTTTAAA

TAGCCAGGCCCCCGATTGCAAAGACATTATAGCGAGCTACCAAAGCCATA

TTCAAACACCTAGATCACTACCACTTCTACACAGGCCACTCGAGCTTGTG

ATCGCACTCCGCTAAGGGGGCGCCTCTTCCTCTTCGTTTCAGTCACAACC

CGCAAACATGACACAAGAATCCCTGTTACTTCTCGACCGTATTGATTCGG

ATGATTCCTACGCGAGCCTGCGGAACGACCAGGAATTCTGGGAGGTGAGT

CGACGAGCAAGCCCGGCGGATCAGGCAGCGTGCTTGCAGATTTGACTTGC

AACGCCCGCATTGTGTCGACGAAGGCTTTTGGCTCCTCTGTCGCTGTCTC

AAGCAGCATCTAACCCTGCGTCGCCGTTTCCATTTGCAGCCGCTGGCCCG

CCGAGCCCTGGAGGAGCTCGGGCTGCCGGTGCCGCCGGTGCTGCGGGTGC

CCGGCGAGAGCACCAACCCCGTACTGGTCGGCGAGCCCGGCCCGGTGATC

AAGCTGTTCGGCGAGCACTGGTGCGGTCCGGAGAGCCTCGCGTCGGAGTC

GGAGGCGTACGCGGTCCTGGCGGACGCCCCGGTGCCGGTGCCCCGCCTCC

TCGGCCGCGGCGAGCTGCGGCCCGGCACCGGAGCCTGGCCGTGGCCCTAC

CTGGTGATGAGCCGGATGACCGGCACCACCTGGCGGTCCGCGATGGACGG

CACGACCGACCGGAACGCGCTGCTCGCCCTGGCCCGCGAACTCGGCCGGG

TGCTCGGCCGGCTGCACAGGGTGCCGCTGACCGGGAACACCGTGCTCACC

CCCCATTCCGAGGTCTTCCCGGAACTGCTGCGGGAACGCCGCGCGGCGAC

CGTCGAGGACCACCGCGGGTGGGGCTACCTCTCGCCCCGGCTGCTGGACC

GCCTGGAGGACTGGCTGCCGGACGTGGACACGCTGCTGGCCGGCCGCGAA

CCCCGGTTCGTCCACGGCGACCTGCACGGGACCAACATCTTCGTGGACCT

GGCCGCGACCGAGGTCACCGGGATCGTCGACTTCACCGACGTCTATGCGG

GAGACTCCCGCTACAGCCTGGTGCAACTGCATCTCAACGCCTTCCGGGGC

GACCGCGAGATCCTGGCCGCGCTGCTCGACGGGGCGCAGTGGAAGCGGAC

CGAGGACTTCGCCCGCGAACTGCTCGCCTTCACCTTCCTGCACGACTTCG

AGGTGTTCGAGGAGACCCCGCTGGATCTCTCCGGCTTCACCGATCCGGAG

GAACTGGCGCAGTTCCTCTGGGGGCCGCCGGACACCGCCCCCGGCGCCTG

ATAAGGATCCCCGCTCCGTGTAAATGGAGGCGCTCGTTGATCTGAGCCTT

GCCCCCTGACGAACGGCGGTGGATGGAAGATACTGCTCTCAAGTGCTGAA

GCGGTAGCTTAGCTCCCCGTTTCGTGCTGATCAGTCTTTTTCAACACGTA

AAAAGCGGAGGAGTTTTGCAATTTTGTTGGTTGTAACGATCCTCCGTTGA

TTTTGGCCTCTTTCTCCATGGGCGGGCTGGGCGTATTTGAAGCGGGTACC

CAGCTTTTGTTCCCTTTAGTGAGGGTTAATTGCGCGCTTGGCGTAATCAT

GGTCATAGCTGTTTCCTGTGTGAAATTGTTATCCGCTCACAATTCCACAC

AACATACGAGCCGGAAGCATAAAGTGTAAAGCCTGGGGTGCCTAATGAGT

GAGCTAACTCACATTAATTGCGTTGCGCTCACTGCCCGCTTTCCAGTCGG

GAAACCTGTCGTGCCAGCTGCATTAATGAATCGGCCAACGCGCGGGGAGA

GGCGGTTTGCGTATTGGGCGCTCTTCCGCTTCCTCGCTCACTGACTCGCT

GCGCTCGGTCGTTCGGCTGCGGCGAGCGGTATCAGCTCACTCAAAGGCGG

TAATACGGTTATCCACAGAATCAGGGGATAACGCAGGAAAGAACATGTGA

GCAAAAGGCCAGCAAAAGGCCAGGAACCGTAAAAAGGCCGCGTTGCTGGC

GTTTTTCCATAGGCTCCGCCCCCCTGACGAGCATCACAAAAATCGACGCT

CAAGTCAGAGGTGGCGAAACCCGACAGGACTATAAAGATACCAGGCGTTT

CCCCCTGGAAGCTCCCTCGTGCGCTCTCCTGTTCCGACCCTGCCGCTTAC

CGGATACCTGTCCGCCTTTCTCCCTTCGGGAAGCGTGGCGCTTTCTCATA

GCTCACGCTGTAGGTATCTCAGTTCGGTGTAGGTCGTTCGCTCCAAGCTG

GGCTGTGTGCACGAACCCCCCGTTCAGCCCGACCGCTGCGCCTTATCCGG

TAACTATCGTCTTGAGTCCAACCCGGTAAGACACGACTTATCGCCACTGG

CAGCAGCCACTGGTAACAGGATTAGCAGAGCGAGGTATGTAGGCGGTGCT

ACAGAGTTCTTGAAGTGGTGGCCTAACTACGGCTACACTAGAAGGACAGT

ATTTGGTATCTGCGCTCTGCTGAAGCCAGTTACCTTCGGAAAAAGAGTTG

GTAGCTCTTGATCCGGCAAACAAACCACCGCTGGTAGCGGTGGTTTTTTT

GTTTGCAAGCAGCAGATTACGCGCAGAAAAAAAGGATCTCAAGAAGATCC

TTTGATCTTTTCTACGGGGTCTGACGCTCAGTGGAACGAAAACTCACGTT

AAGGGATTTTGGTCATGAGATTATCAAAAAGGATCTTCACCTAGATCCTT

TTAAATTAAAAATGAAGTTTTAAATCAATCTAAAGTATATATGAGTAAAC

TTGGTCTGACAG*TTACCAATGCTTAATCAGTGAGGCACCTATCTCAGCGA*

TCTGTCTATTTCGTTCATCCATAGTTGCCTGACTCCCCGTCGTGTAGATA

ACTACGATACGGGAGGGCTTACCATCTGGCCCCAGTGCTGCAATGATACC

GCGAGACCCACGCTCACCGGCTCCAGATTTATCAGCAATAAACCAGCCAG

CCGGAAGGGCCGAGCGCAGAAGTGGTCCTGCAACTTTATCCGCCTCCATC

CAGTCTATTAATTGTTGCCGGGAAGCTAGAGTAAGTAGTTCGCCAGTTAA

TAGTTTGCGCAACGTTGTTGCCATTGCTACAGGCATCGTGGTGTCACGCT

CGTCGTTTGGTATGGCTTCATTCAGCTCCGGTTCCCAACGATCAAGGCGA

GTTACATGATCCCCCATGTTGTGCAAAAAAGCGGTTAGCTCCTTCGGTCC

TCCGATCGTTGTCAGAAGTAAGTTGGCCGCAGTGTTATCACTCATGGTTA

TGGCAGCACTGCATAATTCTCTTACTGTCATGCCATCCGTAAGATGCTTT

TCTGTGACTGGTGAGTACTCAACCAAGTCATTCTGAGAATAGTGTATGCG

GCGACCGAGTTGCTCTTGCCCGGCGTCAATACGGGATAATACCGCGCCAC

ATAGCAGAACTTTAAAAGTGCTCATCATTGGAAAACGTTCTTCGGGGCGA

AAACTCTCAAGGATCTTACCGCTGTTGAGATCCAGTTCGATGTAACCCAC

TCGTGCACCCAACTGATCTTCAGCATCTTTTACTTTCACCAGCGTTTCTG

GGTGAGCAAAAACAGGAAGGCAAAATGCCGCAAAAAAGGGAATAAGGGCG

*ACACGGAAATGTTGAATACTCAT*ACTCTTCCTTTTTCAATATTATTGAAG

CATTTATCAGGGTTATTGTCTCATGAGCGGATACATATTTGAATGTATTT

AGAAAAATAAACAAATAGGGGTTCCGCGCACATTTCCCCGAAAAGTGCCA

C

Supplementary Figure 7 | Complete sequence of pHD-AEQ2 bearing the aequorin cassette. Sequences in bold letters represent the tandem promoter. It is followed by the codon-optimized *apo-aequorin* represented in bold and underlined letters. The *RACK1* 3'-UTR is represented in italic letters. The *Aph7´´* (hygromycin B) resistance cassette is underlined and the ampicillin resistance cassette for the bacterial selection is represented in italics and underlined letters.

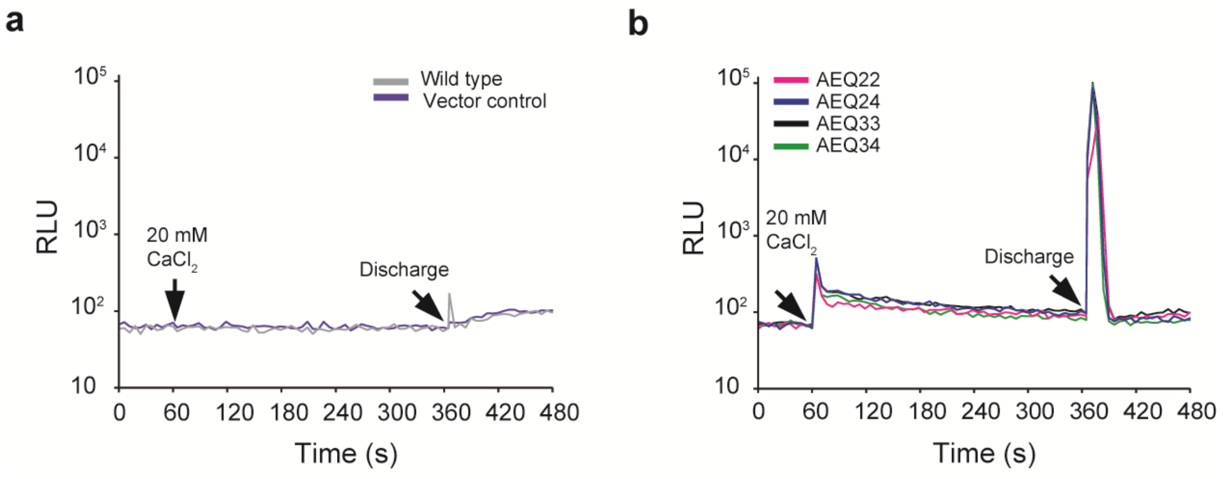

Supplementary Figure 8 | Determination of the Ca^{2+} concentration by measuring discharge after calcium chloride treatment. (a, b) Maximum luminescence of 20 mM $CaCl_2$ treated cells (see Fig. 3c) was obtained by adding 100 µL of 2 M $CaCl_2$ in 10% ethanol (black arrowhead labeled with discharge at the right side) to coelenterazine treated cells [wild type and vector control in (a); AEQ22, AEQ24, AEQ33 and AEQ34 in (b)] after completion of Ca^{2+} measurement. Each line in the figure represents the mean of experiments performed with three biological replicates and includes three technical replicates per biological replicate. All experiments were replicated twice. RLU, relative light units.

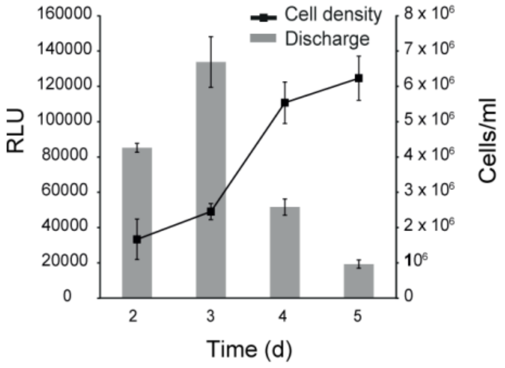

Supplementary Figure 9 | Aequorin activity as a function of growth phase. Freshly inoculated cells were grown up to five days. Samples from days two, three, four and five were taken for measurement. For every sample measured, the cell density was adjusted to 4 x 10^6 cells ml^{-1}, by either concentrating or diluting the cultures. Maximum luminescence (discharge) was determined as in (Supplementary Fig. 8). Each data point represents the mean of three biological replicates, and each biological replicate includes three technical replicates. The experiment was performed once. Error bars represent the standard deviation. RLU, relative light units.

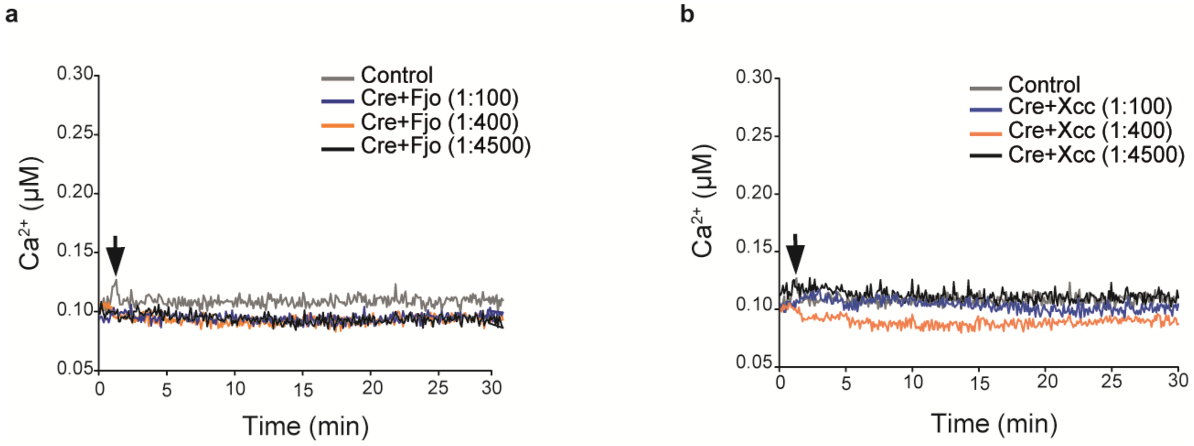

Supplementary Figure 10 | *F. johnsoniae* (a) and *X. campestris* (b) do not elicit a Ca^{2+} signal in coculture. Time course of cytosolic Ca^{2+} concentrations after addition of bacteria to AEQ34 cells. For the Ca^{2+} measurements, the bacterial cell density was adjusted to the indicated cell ratios. As control, LB broth was added (black arrowhead). Each line in the graph represents the mean of three biological replicates, and each biological replicate includes three technical replicates. All experiments were replicated twice.

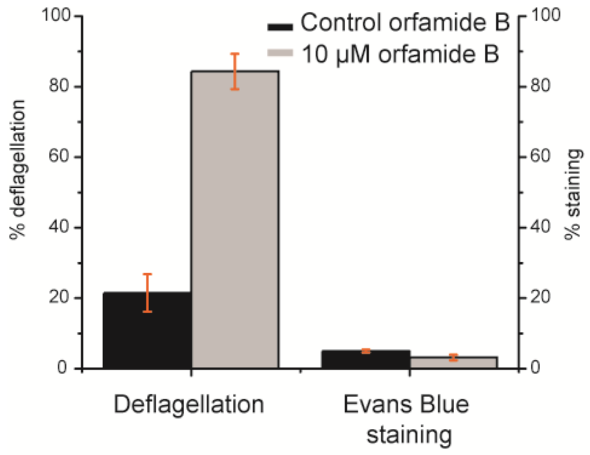

Supplementary Figure 11 | Orfamide B deflagellates *C. reinhardtii*, but does not cause a major permeabilization of cells. Deflagellation and Evans blue staining in wild type upon treatment with orfamide B at the indicated concentrations. The experiment was performed with three biological replicates and replicated twice.

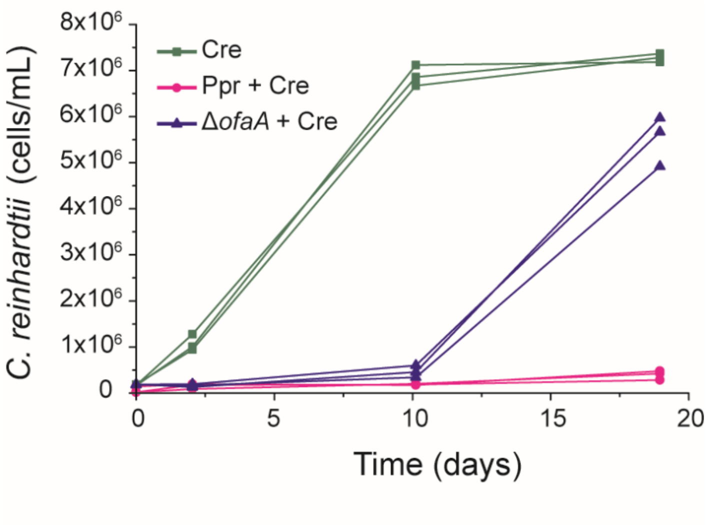

Supplementary Figure 12 | Growth curves of *C. reinhardtii* in coculture with *P. protegens* or the Δ*ofaA* mutant and alone. The growth of *C. reinhardtii* cultured together with wild-type *P. protegens* (Ppr + Cre) or the Δ*ofaA* mutant (Δ*ofaA* + Cre) is compared to axenic algal cultures (Cre; see also Fig. 4g). A 1:100 ratio of algae to bacteria was used for inoculation with an initial concentration of 10^5 algal cells mL^{-1}. The experiment was performed once with three biological replicates.

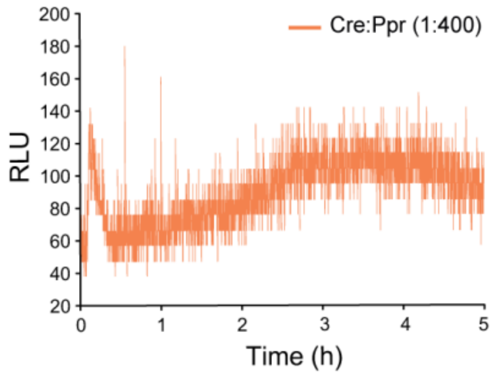

Supplementary Figure 13 | *P. protegens* elicits also on long-term a Ca^{2+} signal in coculture. The assay was performed as described in Fig. 4a, but the measurement was prolonged for 5 h. The data are represented in relative light units (RLU) as long-term incubation with *P. protegens* causes the depletion of coelenterazine loaded aequorin, which impedes an accurate conversion to absolute Ca^{2+} concentrations. The line in the graph represents the mean of three biological replicates, and each biological replicate includes three technical replicates. The experiment was replicated twice.

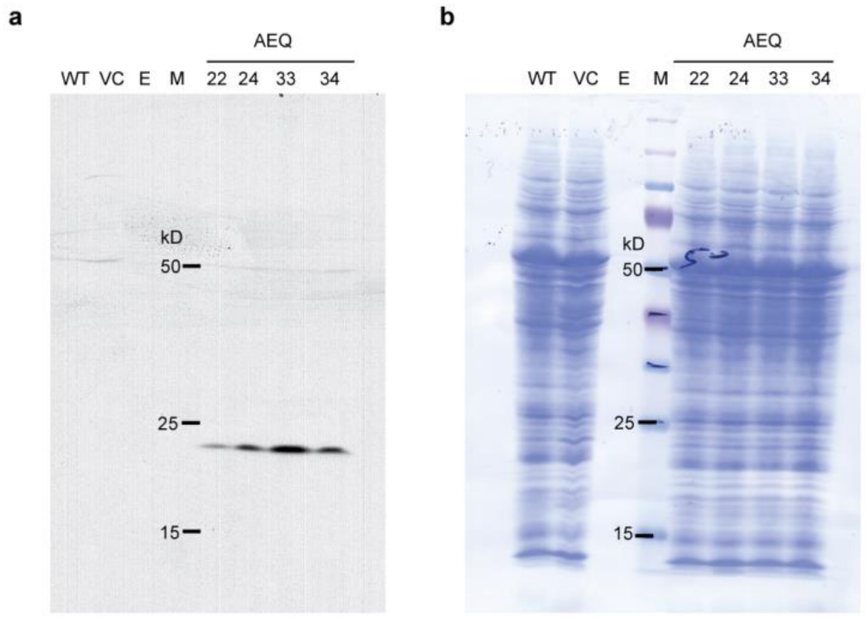

Supplementary Figure 14 | Full immunoblot and stained membrane as presented in Figure 3b. (a) Immunoblot showing the expression of apo-aequorin (22 kD) in transgenic lines AEQ22, AEQ24, AEQ33 and AEQ34 compared to wild type (WT) and a vector control (VC). **(b)** As a loading control, the Coomassie-stained PVDF membrane was used. E: empty lane; M: Molecular weight marker.

Supplementary References:

1 Harris, E.H. Ed., The *Chlamydomonas* Sourcebook (CA: Academic Press San Diego, 1989).

2 S. Ashcroft & C. Pereira, Practical statistics for the biological sciences, Palgrave Macmillan, Basingstoke UK (2003).

3 Yang, J. Y. *et al.* Primer on agar-based microbial imaging mass spectrometry. *J. Bacteriol.* **194**, 6023-6028 (2012).

4 Gross, H. *et al.* The genomisotopic approach: a systematic method to isolate products of orphan biosynthetic gene clusters. *Chem. Biol.* **14**, 53-63 (2007).

5 Kumar, S., Stecher, G. & Tamura, K. MEGA7: Molecular evolutionary genetics analysis version 7.0 for bigger datasets. *Mol. Biol. Evol.* **33**, 1870-1874 (2016).
