# Supplementary material for: Antagonistic bacteria disrupt calcium homeostasis and immobilize algal cells
Source: Nat Commun. 2017 Nov 24;8:1756. doi: 10.1038/s41467-017-01547-8 (PMC5701020; doi:10.1038/s41467-017-01547-8)
Supplement: Supplementary file 2 — Description of Additional Supplementary Files [file 41467_2017_1547_MOESM2_ESM.pdf]

Supplementary Information

Supplementary Movie 1: Swarming of *P. protegens* cells around *C. reinhardtii* cells in mixed culture.

Supplementary Movie 2: *C. reinhardtii* movement before treatment with orfamide A.

Supplementary Movie 3: *C. reinhardtii* movement after treatment with orfamide A.

Supplementary Movie 4: *C. reinhardtii* movement before treatment with methanol (control).

Supplementary Movie 5: *C. reinhardtii* movement after methanol treatment (control).

Supplementary Movie 6: *Chlamydomonas* sp. SAG 25.89 movement before treatment with orfamide A.

Supplementary Movie 7: *Chlamydomonas* sp. SAG 25.89 movement after treatment with orfamide A.

Supplementary Movie 8: *H. pluvialis* movement before treatment with orfamide A.

Supplementary Movie 9: *H. pluvialis* movement after treatment with orfamide A.

Supplementary Movie 10: *G. pectorale* movement before treatment with orfamide A.

Supplementary Movie 11: *G. pectorale* movement after treatment with orfamide A.

Supplementary Movie 12: *P. minor* movement before treatment with orfamide A.

Supplementary Movie 13: *P. minor* movement after treatment with orfamide A.

Supplementary Movie 14: *E. gracilis* movement before treatment with orfamide A.

Supplementary Movie 15: *E. gracilis* movement after treatment with orfamide A.
